# HDAC6 inhibition by ITF3756 modulates PD-L1 expression and monocyte phenotype: insights for a promising immune checkpoint blockade co-treatment therapy

**DOI:** 10.3389/fimmu.2025.1546939

**Published:** 2025-05-13

**Authors:** Valeria Spadotto, Chiara Ripamonti, Andrea Ghiroldi, Elisabetta Galbiati, Pietro Pozzi, Roberta Noberini, Tiziana Bonaldi, Christian Steinkühler, Gianluca Fossati

**Affiliations:** ^1^ New Drug Incubator Department, Italfarmaco Group, Milan, Italy; ^2^ Preclinical Drug Development Department, Italfarmaco Group, Milan, Italy; ^3^ Department of Experimental Oncology, IEO European Institute of Oncology IRCCS, Milan, Italy; ^4^ Department of Oncology and Hematology-Oncology (DIPO), University of Milan, Milan, Italy

**Keywords:** HDAC6, monocytes, immuno-checkpoints, TNF-α, dendritic cells

## Abstract

**Introduction:**

Tumor immunotherapy has revolutionized cancer treatment, particularly through the use of immune checkpoint inhibitors targeting the PD-L1/PD-1 axis. While PD-L1 expression on tumor cells is an established predictive biomarker for therapeutic response, emerging evidence highlights the importance of PD-L1 expression on myeloid cells, both in the periphery and within the tumor microenvironment (TME). This study explores the immunomodulatory effects of the selective HDAC6 inhibitor ITF3756 on monocytes and dendritic cells (DCs).

**Methods:**

Monocytes were stimulated with the pro-inflammatory cytokine TNF-α and treated with ITF3756. PD-L1 and CD40 expression levels were assessed by flow cytometry. Transcriptomic and proteomic analyses were performed to characterize changes in gene and protein expression profiles. T cell proliferation was evaluated in co-culture assays. Additionally, the impact of ITF3756 was assessed in an in vivo murine model of colon cancer.

**Results:**

ITF3756 effectively downregulated PD-L1 expression in TNF-α-activated monocytes and enhanced their costimulatory capacity by increasing CD40 expression. Transcriptomic and proteomic analyses revealed that ITF3756 counteracted TNF-α pathway activation and downregulated multiple inhibitory immune checkpoint molecules, promoting a less immunosuppressive phenotype. In co-culture assays, ITF3756-treated monocytes and DCs significantly enhanced T cell proliferation. In vivo, ITF3756 treatment led to reduced tumor growth in a colon cancer model.

**Discussion:**

These findings demonstrate that selective HDAC6 inhibition by ITF3756 modulates myeloid cell functionality by diminishing inhibitory signals and promoting T cell activation. Thus, ITF3756 represents a promising immunomodulatory agent that could enhance the efficacy of immune checkpoint blockade in cancer immunotherapy.

## Introduction

Tumor immunotherapy has become the standard of care, in the treatment of a variety of tumors. Stimulation of tumor immune response with antibodies directed to immune checkpoint molecules gives in a fraction of patients, impressive results with long-lasting tumor regressions (1, 2).

The most prominent immunotherapy target is the programmed death ligand-1/programed death-1 (PD-L1/PD1) axis, and the inhibition of this pathway with specific antibodies has shown clinical efficacy in many tumors, with an enhancement of T cell responses and antitumor activity (2, 3). Initially, it was considered that the expression of PD-L1 on tumor cells was essential in the response to anti-PD-L1 antibodies. Indeed, the expression of PD-L1 was used as a predictive biomarker, since patients with an elevated PD-L1 expression on cancer cells had a better response to the treatment. However, further studies have shown that also patients with reduced PD-L1 expression on tumor cells can respond to the therapy, indicating that other cells within the tumor microenvironment (TME) are involved in the antitumor activity mediated by the inhibitors of PD-1PD-L1 axis (4). Several studies have recently highlighted that PD-L1 expression on innate immune cells such as macrophages, dendritic cells (DC) and monocytes, both in the TME and outside the tumor tissue, is essential for an effective PD-L1 and PD-1 blockade (5, 6). Lin et al. (5) reported that anti-PD-L1 treatment failed to control tumor growth and immune response in PD-L1 KO mice bearing tumor cells expressing higher level of PD-L1. On the contrary, in mice with PD-L1-negative tumor, but with antigen presenting cells (APC) expressing PD-L1, the immune response mediated by anti-PD-L1 therapy was effective. These conclusions were also supported by Tang et al. (6). They showed in three different tumor models (MC38, A20 and EG7) that PD-L1 expression in tumor cells was dispensable for the effective response to the anti-PD-L1 therapy, while its expression in myeloid cells was crucial since PD-L1 expression on these cells actively participated to the suppression of T cell function. Both papers suggest that PD-L1 expression on tumor cells is not essential for an efficient response to anti-PD-L1 therapy, and this could explain why also some patients negative for PD-L1 in the tumor respond to PD-L1/PD-1 blockade. Moreover, Tang and colleagues proposed the possibility that some patients could express higher levels of PD-L1 in the myeloid compartment outside the TME and that this might explain the overall response of these patients to therapy. Additionally, PD-L1 expression on myeloid cells in ovarian cancer and melanoma patients correlated with the success of the therapy with either anti–PD-L1 alone or in combination with an anti–CTLA-4 (5).

Myeloid cells are a major component of TME and play a key role in shaping the immune tolerant milieu that is crucial for cancer growth. Interactions between myeloid cells and tumor cells can support tumor growth. Immature monocytes are, for example, precursors of tumor-associated macrophages (TAM), which have an immune-suppressive phenotype, and monocytes themselves can promote immune suppression upregulating PD-L1 expression. In human and mice, myeloid cells in the TME express high levels of functional PD-L1 and local release of cytokines such as TNF-α and IFN-γ can increase its expression (7, 8). Moreover, monocytes can differentiate into DCs which efficiently present tumor neo antigens to stimulate the anti-tumor T cell response. However, cancer cells can dampen the activation of T cells by DCs in various ways, such as by inducing the expression of PD-L1 on DCs surface (9).

These observations support the idea that the specific modulation of PD-L1 in myeloid cells could be exploited as a possible therapy to enhance the immune response induced by checkpoints blockade. In this context, it is known that histone deacetylases inhibitors (HDACi) can modulate PD-L1 expression on tumor cells as well as on immune cells (10). Histone deacetylases (HDACs) are a family of epigenetic regulatory enzymes originally discovered for their ability to remove acetyl groups from the lysine residues of histone tails. The zinc-dependent, or classical HDACs are divided into four classes: class I (HDAC1, HDAC2, HDAC3, HDAC8), class IIA (HDAC4, HDAC5, HDAC7, HDAC9), class IIB (HDAC6 and HDAC10) and class IV (HDAC11). These enzymes are now recognized to have a broader biological function since they can remove acyl groups from the side chain of lysine of histones and several other proteins (11). Some pan-HDACi are FDA-approved for the treatment of hematologic malignancies, and, more recently for the treatment of Duchenne muscular dystrophy (12, 13). However, the therapeutic index of these inhibitors is narrow, and dose-limiting toxicities, such as thrombocytopenia, are observed in the clinic (14, 15). Subtype-selective HDAC inhibitors bear the potential of maintaining a therapeutic capability while decreasing toxicities. Among the Zinc-dependent HDAC, HDAC6 is a peculiar enzyme with a recognized involvement in tumor growth and development and in the modulation of the immune functions (16). Interestingly, HDAC6 KO mice are viable and fertile indicating that this isozyme is an ideal pharmacological target to modulate tumor and immune functions with a favorable therapeutic index. To verify this assumption, selective HDAC6i have been recently developed and evaluated in clinical trials. As regards immune checkpoints, the use of non-selective HDACi is known to increase the expression of PD-L1 on the cell surface of tumor cells (17, 18), while selective HDAC6i have shown the opposite effect, downregulating the expression of PD-L1. In particular, the group of Villagra highlighted the role of HDAC6 in PD-L1 modulation in a melanoma cells line and reported that Nexturastat, a selective HDAC6i, counteracted the IFN-γ induced up-regulation of PD-L1 on tumor cells, thus increasing the antitumor immune response of anti PD-L1 treatment (19). Besides PD-L1 modulation on tumor cell lines, it has been recently reported that the HDAC6i inhibitor ACY-241, in combination with oxaliplatin, modulates PD-L1 level on TAMs in a mouse model of non-small cell lung cancer (NSCLC) (20). In a different preclinical mouse model, Ray et al. observed that ACY-241 significantly decreases PD-L1 expression on plasmacytoid dendritic cells (pDCs), reducing PD-L1/PD-1-mediated NK and T suppression and thereby enhancing the cells cytotoxicity (21). The literature already reports the use of small molecules, such as HDACi, alone or in combination with PD-1/PD-L1 inhibitors, as cancer therapy in preclinical models and clinical trials (20–22). Combinations of HDACi, such as ACY-241 (a moderately selective HDAC6 inhibitor) or Entinostat (a class I inhibitor) with anti PD-1/PD-L1 antibodies have been tested in several clinical trials to treat various cancers (e.g. clinical trial reference: NCT02635061, NCT02915523, NCT02697630) (22). In recent years, increasing attention has been given to the development of small-molecule-based PD-1/PD-L1 inhibitors, aiming to overcome the limitations of antibody-based therapies, including serious side effects, long half-lives, complex manufacturing processes, and high costs. Several small molecules targeting PD-1/PD-L1 axis are currently under investigation, both in clinical trials (e.g. MAX-10181, INCB086550, IMMH-010) and in preclinical models (e.g. BMS series of compounds, particularly BMS-202) (22). Besides to direct inhibitors of the PD-1/PD-L1 axis, other strategies have been explored, particularly targeting transcription and translation pathways, such as the bromodomain inhibitor, JQ1 and the MNK1 inhibitor, eFT508, as well as promoting the PD-L1 degradation with Curcumin (23). Despite the numerous benefits of small molecule inhibitors, including reduced immunogenicity, improved tissue penetration, lower production costs, and greater flexibility in pharmacokinetics optimization, their target affinity is generally lower than that of antibody-based drugs. In addition, small molecules may be more prone to off-target effects, which may reduce therapeutic efficacy and cause unknown toxicities (23). For this reason, the development and characterization of new small molecules targeting the PD-1/PD-L1 axis remains a critical research area in this emerging field.

Considering the effect of HDAC6i on the immune-checkpoint inhibitors on certain myeloid cells and considering that PD-L1 modulation of immune system cells seems to be essential for the response to PD-1/PD-L1 blockade, we asked whether our selective HDAC6i ITF3756 (24), currently in phase I clinical trial in patients with advanced solid tumors, could reduce the PD-L1 expression in an *in vitro* model of monocytes stimulated with a pro-inflammatory cytokine. In the TME the level of IFN-γ is increased after PD-1 immune checkpoint blockade, and this caused an up-regulation of PD-L1 in tumor cells (19). However, besides IFN-γ, also other cytokines produced in the TME have been found to upregulate PD-L1 expression and it was reported that endogenous production of TNF-α in the TME is required for the upregulation of PD-L1 on monocytes and TAMs (7). Therefore, in our *in vitro* model, we decided to stimulate monocytes from human healthy donors with TNF-α and to treat them with ITF3756.

We show that ITF3756 can reduce the immunosuppressive phenotype of TNF-α stimulated monocytes by downmodulating PD-L1, while promoting their costimulatory capacity by inducing CD40 expression. Transcriptomic and proteomic analyses shed light on the global effect of ITF3756 on TNF-α-stimulated monocytes, revealing that the HDAC6 inhibitor strongly reduces TNF-α pathway activation and promotes a less immunosuppressive phenotype by downregulating not only PD-L1 expression, but also several other immune checkpoints. Moreover, functional assays performed by co-culturing monocytes with allogenic T cells, show that the phenotype acquired by monocytes treated with ITF3756 enhance T cells proliferation compared to untreated TNF-α-stimulated monocytes, suggesting the induction of an improved T cell activation status.

Monocytes can also differentiate into DC, which regulate the adaptive immune response in the TME. With the goal of further understanding the effect of ITF3756 on myeloid cells, we also investigated the impact of our HDAC6i on the DC activation status and on their capacity to increase T cell proliferation in a co-culture model. The results confirmed that ITF3756 enhances the APCs phenotype of immature (iDCs) and mature (mDCs), strengthening the effect on allogeneic T cells proliferation.

Based on data collected *in vitro*, we assessed ITF3756 efficacy *in vivo* in a murine model of colon carcinoma. In this model, ITF3756 demonstrated an anti-tumoral activity, effectively reducing tumor growth in a dose response manner.

Our data indicate that HDAC6 inhibition reduces PD-L1 expression in monocytes stimulated with the pro-inflammatory cytokine TNF-α. Overall, the results obtained indicate that ITF3756 makes myeloid cells less immunosuppressive, acting on multiple immune checkpoint inhibitors and less pro-inflammatory, dampening TNF-α pathway but, at the same time, active and functional in promoting T cell proliferation. The *in vivo* results show an anti-tumoral effect of ITF3756. These results suggest the use of ITF3756 as an immune modulating agent targeted to enhance the antitumor response of immune checkpoint blockade.

## Materials and methods

### *In vivo* study

This study was performed using 6 weeks old female balb/c mice, obtained from Charles River Italia (Calco, LC). Mice were housed in Italfarmaco’s facility under pathogen free conditions in room with controlled conditions (temperature, 22 ± 2°C, relative humidity, 50 ± 10% and a 12 hour light/dark cycle) and free access to food and water. After 10 days of acclimatization mice were submitted to tumor cells injection. The *in vivo* study was approved by the internal Animal Care and Use Committee and was performed in agreement with the Italian legislation D.Lgs 26/2014.

### Reagents

ITF3756 was synthesized by Italfarmaco Medicinal Chemistry Department. ITF3756 was weighed and dissolved in DMSO (Sigma Aldrich) at 20mM and used as stock solution. Working solutions were made by diluting the stock solutions in complete medium (RPMI 1640 (Biochrom) + 10% Fetal Bovine Serum (FBS) (Cytiva) and penicillin-streptomycin (Sigma Aldrich).

### Monocytes purification and activation

Peripheral blood mononuclear cells (PBMCs) used for the experiments were obtained from buffy coats of healthy volunteers and separated over a Ficoll-Hypaque gradient (Biochrom). All samples tested negative for transmissible diseases as required for blood transfusion.

Monocytes were purified from 100x10^6^ human PBMCs by negative selection using Pan Monocytes Isolation Kit (Miltenyi) or by positive selection using CD14 Microbeads (Miltenyi) following manufacturers’ instructions. Purified monocytes (1x10^6^/ml) were pre-treated for 2h with ITF3756 1μM in 12 well plate in 1–2 ml final volume in RPMI medium supplemented with 10% Fetal Bovine Serum (FBS). The cells were then stimulated with TNF-α (Peprotech) (100ng/ml) overnight (ON). After ON incubation, the cells were analyzed for the expression of CD40 and PD-L1 by flow cytometry.

PBMC collected from cancer patients (Colorectal cancer and Brest cancer) were acquired by TEBU-BIO SRL, and patients’ data are reported in **Supplementary Figures 2A, B**. PD-L1 expression was assessed on PBMC instead of purified monocytes due to the small number of cells in the samples. PBMC were treated and analyzed as reported above.

For washout experiments, purified monocytes were stimulated with TNF-α (100ng/ml) for 2h, 4h, 6h and 18h to investigate the minimum exposure time to obtain an effective modulation of PD-L1. Subsequently, the same type of experiment was conducted adding ITF3756 1μM to TNF-α stimulated monocyte for 2h, 4h, 6h, 10h and 18h. After each time point cells were centrifuged and the medium was removed, new medium without TNF-α and ITF3756 was added to the cells. After 18h, cells from all conditions were analyzed for the expression of PD-L1 by flow cytometry.

For genes expression analysis, a time course experiment was performed by pre-treating the monocytes for 2h with ITF3756 1μM followed by a stimulation with TNF-α (100ng/ml) for 1, 2 and 4h. After incubation, the cells were collected and stored at -80°C for subsequent qPCR analysis or RNAseq analysis.

For signaling pathway analysis, purified monocytes were pretreated with ITF3756 1μM for 2h and stimulated with TNF-α (100ng/ml) for 15 minutes. After incubation, the cells were collected and analyzed for p65 protein phosphorylation by flow cytometry.

For mass spectrometry-based proteomics analysis, monocytes were purified from 200x10^6^ human PBMC by negative selection using the Pan Monocytes Isolation Kit (Miltenyi). Cells were then pre-treated for 1h with ITF3756 1μM and then stimulated with TNF-α (100ng/ml) (Peprotech). After 18h, treated cells were collected, washed with PBS and stored at -20°C before subsequent analysis. For each sample, 3 technical replicates were performed. Samples from 3 different donors were analyzed.

### Differentiation and activation of purified monocytes with GM-CSF and IL-4

Monocytes were purified by positive selection using CD14 Microbeads (Miltenyi). Purified monocytes (1x10^6^/ml) were treated or not treated with ITF3756 (1.0-0.5μM) and at the same time stimulated with GM-CSF (Peprotech) (50ng/ml) and IL-4 (Peprotech) (10ng/ml) for 5 days in 12-wells plate in 1-2ml final volume in RPMI medium supplemented with 10% FBS. After 5 days of incubation, immature DCs were obtained. In some experiments, after differentiation, immature DCs were pre-treated with ITF3756 for 2h and then stimulated with LPS (1μg/ml) for 18h to obtain mature dendritic cells. After differentiation or LPS activation, cells were analyzed for PD-L1 and CD86 expression by flow cytometry. An aliquot of cells was collected and stored at -80°C for subsequent qPCR analysis.

### Mixed lymphocytes reaction

Purified monocytes pre-treated or not treated with ITF3756 1μM and stimulated ON with TNF-α (100ng/ml) were used in a Mixed Lymphocytes Reaction (MLR) assay. After ON incubation, monocytes were washed and co-cultured at different ratios (1:2,1:4,1:8,1:16 monocytes:T cells) with allogeneic T cells (1X10^5^ CD3 T cells, purified by positive selection using CD3 Microbeads (Miltenyi)) in 96-well plate in 200μl final volume. T cells were previously stained with 2 μM of CarboxyFluoroscein Succinimidyl Ester (CFSE) in PBS at 37°C for 10 minutes, then PBS-10% FBS was added to the cells and incubated at 4°C for 5 minutes. After incubation, cells were washed and co-cultured with monocytes. Proliferation was determined by CFSE dilution on day 6. In some experiments, antibody anti-PD-L1 (2.5μg/ml, ThermoFisher Scientific) was added to monocytes-T cell co-culture. Samples were acquired using the flow cytometer BD FACSVerse and data were analyzed using the FlowJo software.

ITF3756 treated or not treated immature DCs were washed and co-cultured with CFSE-labelled allogeneic T cells (1x10^5^, purified CD3 T cells, ratio iDC: T cells 1:10) in 96-well plate in 200μl final volume. T-cell proliferation was quantified after 5 days of co-culture by CFSE dilution. Samples were acquired using the flow cytometer BD FACSVerse and data were analyzed using the BD FACSuite software.

### Flow cytometry

Purified monocytes, Human PBMC and DCs were treated with human FcR blocking reagent (Miltenyi) and stained for 20 minutes at RT with the following fluorochrome-conjugated antibodies: for monocytes and PBMC analysis, anti-CD14 PE (clone MP9,BD Bioscience), anti-PD-L1 BV415 (BD Bioscience), anti PD-L1 PE (eBioscience), and anti-CD40 PE (Miltenyi), while for DC, anti-CD86 APC (Miltenyi) and anti-PD-L1 PE (eBioscience). PD-L1 and CD40 expression was analyzed in CD14+ gate (or monocytes gate identified by forward and side scatter).

For protein phosphorylation analysis intracellular staining was performed. Cells were fixed with BD Cytofix buffer (BD Bioscience) for 10min at 37°C. After incubation cells were washed and permeabilized with BD PhosFlow Perm Buffer III (BD Bioscience) on ice for 30min, washed twice with PBS-0.1% FBS and incubated with anti-phosphorylated p65 Ser536 Alexafluor488 (Cell Signaling) antibody and human FcR blocking for 1h at RT. Samples were washed after antibodies incubation and fluorescence was acquired using the flow cytometer BD FACSVerse. Data were analyzed using the BD FACSuite software.

### 7AAD

ITF3756 toxicity was evaluated at different concentrations, after treatment ON, by 7AAD (7-aminoactinomycin D) staining, following manufacturer’s instructions. Samples were acquired using the flow cytometer BD FACSVerse and data were analyzed using the BD FACSuite software.

### RNA extraction, cDNA synthesis and real time PCR

Cells were collected by centrifugation and total RNA was extracted with Trizol reagent (ThermoFisher Scientific), following manufacturer’s instructions. RNA concentration and estimation of purity were determined by absorbance reading at 260 and 280 nm with NanoDrop ND-1000 Spectrophotometer (Thermo Scientific). cDNA was synthesized by reverse transcription using the Advantage RT-for-PCR Kit (Clontech) and used to perform SYBR Green based qPCR analysis.

All the qPCR amplifications were achieved by Step One Plus instrument (ThermoFisher Scientific). The data were analyzed using the double delta Ct (DDCt) method. qPCR primers PrimeTime Predesigned qPCR Assays (IDT or SABioscience, Qiagen) used were the following:

*CD40*: Hs.PT.58.3418957

*PD-L1* (CD276): Hs.PT.58.1389336

*CD86*: Hs.PT.58.21526437

*HDAC6*: Hs.PT.58.27574437

*β-actin*: PPH00073G

### Transcriptomics analysis

RNA was extracted from treated monocytes with Trizol reagent according to the manufacturer’s instructions, concentration and purity estimated as above and stored at -80°C. RNA quality was confirmed using a 2100 Byoanalyzer (Agilent), and only RNAs with an RIN (RNA integrity number) >7 were included in the analysis. Transcriptomic analyses were conducted in collaboration with CRS4 (Pula, Cagliari, Italy) on 4 different donors. To generate RNA libraries, the TruSeq stranded mRNA protocol was performed, starting from a total RNA of 200 ng. The RNA libraries were then sequenced on an Illumina NextSeq2000 sequencer, generating 30 M paired-end reads of 75 nucleotides in length for each run. The transcriptomics sequencing data were pre-processed to remove adapters and low-quality fragment ends with Cutadapt (v4.1) and mapped to the Ensembl 98 Human Genome build (GRCh38.p13) using STAR 2 in a splicing-aware setting, adjusting the splicing junction overhang parameter (–sjdbOverhang) to match the sample library read length (75 base-pairs). The genomic alignments in transcriptomic coordinates generated using STAR were analyzed using the RSEM suite to obtain gene expression levels genome wide. The differential expression analysis was performed using DESeq2 (25) which was focused on protein-coding genes, with detectable expression (i.e., gene read counts >0) in at least 50% of the samples, which yielded 14,480 protein coding genes. The design formula included factors for gender, sample, and treatment. The complete design formula was: ~ gender + subject + treatment. Threshold setting for differential gene expression analysis were Log2FoldChange=+/-0.5, p-value adjusted=0.05. Pathway enrichment analysis was performed with the EnrichR (26) package on R, using all the detectable genes as background. Transcriptional factor activity was inferred using DecoupleR package (27).

### Liquid chromatography coupled to tandem mass spectrometry analysis

Monocytes pellets obtained from 3 different donors (see Monocytes purification and activation) were digested to obtain the peptide mixtures for LC-MSMS analysis using the PreOmics iST sample preparation kit, following the manufacturer’s guidelines. Peptide mixtures were separated by reversed-phase nano-liquid chromatography on an EASY-Spray column (Thermo Fisher Scientific), 25-cm long (inner diameter 75 µm, PepMap C18, 2 µm particles), which was connected online to a Q Exactive Plus instrument (Thermo Fisher Scientific) through an EASY-Spray™ Ion Source (Thermo Fisher Scientific). Solvent A was 0.1% formic acid (FA) in ddH_2_O and solvent B was 80% ACN plus 0.1% FA. Peptides were injected in an aqueous 1% trifluoroacetic Acid (TFA) solution at a flow rate of 500 nL/min and were separated with a 5%–65% gradient (70 min 5-20%, 15 min 20-30%, 5 min 30-65%), at a flow rate of 300 nL/min. The Q Exactive Plus instrument was operated in the data-dependent acquisition (DDA) mode. Survey full scan MS spectra (*m*/*z* 375–1650) were analyzed in the Orbitrap detector with a resolution of 70,000 at *m*/*z* 200. The 15 most intense peptide ions with charge states comprised between 2 and 7 were sequentially isolated to a MS1 target value of 3×10^6^ and fragmented by HCD with a normalized collision energy setting of 28%. The maximum allowed ion accumulation times were 20 msec for full scans and 100 msec for MS/MS, and the target value for MS/MS was 1×10^5^. The dynamic exclusion time was 20 sec.

The acquired raw data were analyzed using the integrated MaxQuant software v.1.6.2.3 [Max Planck Institute of Biochemistry (28)] and the Uniprot HUMAN (181029) databases. Enzyme specificity was set to trypsin and two missed cleavages were allowed. Methionine oxidation and N-terminal acetylation were included as variable modifications and the FDR was set to 1%, both at the protein and peptide level. The label-free software MaxLFQ (29) was activated, with the “match between runs” feature (match from and to, matching time window=2 min). The mass spectrometry proteomics data have been deposited to the ProteomeXchange Consortium (30) via the PRIDE partner repository with the dataset identifiers PXD057931. The “protein groups” MaxQuant output file was analyzed using Perseus (31), using LFQ averages of two technical replicates. No imputation was used, and the data were filtered to have at least 75% of valid values in at least one group. Protein with an FDR<0.05 were considered differential expressed.

Differential expression analysis was performed with the limma package on R. The design formula used for the linear model fit was: ~ gender + subject + treatment. Pathway enrichment analysis was performed with the EnrichR package on R, using all the detected proteins as background.

### CT26 colon cancer model

Adult BALB/c mice were subcutaneously injected with 1x10^6^ CT26 tumor cells (diluted to 200μl with phosphate-buffered saline). When the tumor nodule was palpable in at least 90% of the animals, the mice were randomized in the experimental groups (18 mice per group), and drugs administration was started. Treatments were done by oral gavage using a stainless steel bulb tipped gavage needle attached to a 1mL syringe. The following treatments were administrated: vehicle (5% DMSO in H2O/PEG400 1:1(10 ml/Kg, three times a day (TID)), ITF3756 25 mg/kg twice a day (BID), ITF3756 25 mg/kg three times a day (TID), ITF3756 50mg/kg once a day (QD), ITF3756 50mg/kg twice a day (BID), ITF3756 50 mg/kg three times a day (TID). Mice were evaluated for tumor growth every two or three days. Tumor nodules were measured using a caliper and relative volume was obtained using the following formula defining an ellipsoid: Volume (mm3) = (D x d2)/2 where D = larger diameter of the nodule e d = smaller diameter of the nodule (32, 33). The weight of the tumor nodule was calculated assuming the density value of 1g/mL. The data were expressed as mean ± SE.

### Additional statistical analyses

All data were analyzed using the GraphPad Prism 10 software. RM one-way ANOVA followed by Dunnett’s *post hoc* multiple comparison test was used for statistical analyses. For the *in vivo* experiment two-way ANOVA followed by Tukey’s multiple comparisons test was used for the statistical analysis. Where indicated, paired, two-tailed, t-test was used to determine statistical significance between control and a specific treatment group. P-values ≤ 0.05 were considered statistically significant, otherwise differently specified. When not specified, the analysis gave a non-statistically significant difference.

## Results

### ITF3756 modulates PD-L1 and CD40 expression in TNF-α stimulated human monocytes

The tumor microenvironment is a complex biological milieu that the tumor cells shape to maintain tumor growth. Thus, specific inflammatory conditions are generated by the tumor to maintain an immunosuppressive milieu. Increased activity of the PD-1/PD-L1 axis is one of the key pathways to suppress the antitumor immune response. We investigated whether our HDAC6i, ITF3756, could reduce PD-L1 expression in an *in vitro* model of TNF-α stimulated human monocytes. Furthermore, we also hypothesized that besides the downregulation of this immunosuppressive pathway, HDAC6 inhibition could also increase the costimulatory activity of myeloid cells by upregulating the expression of costimulatory molecules such as CD40.

**Figure 1:**
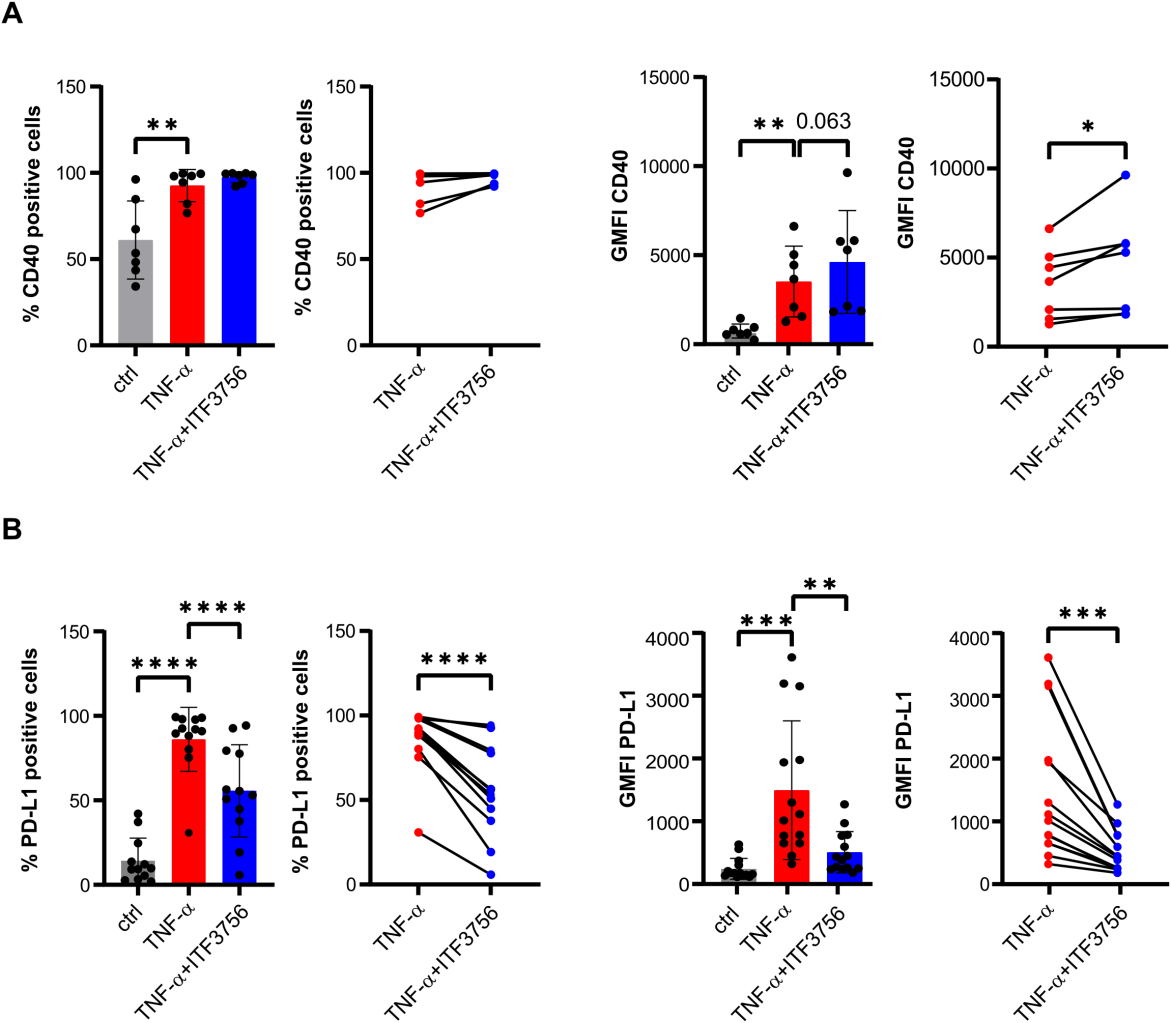
ITF3756 treatment significantly upregulates CD40 and downregulates PD-L1. Human purified monocytes were treated for 2h with ITF3756 (1μM) and then stimulated with TNF-α (100ng/ml) ON. **(A)** Percentage of CD40 positive cells and expression of CD40 measured as the fluorescence intensity geometric mean (GMFI). For both the percentage of CD40 positive cells and for CD40 GFMI, the graphs on the left show the analysis of CD40 in all experimental conditions where statistical analysis was performed using RM one-way ANOVA followed by Dunnetts multiple comparison test, while the graphs on the right show the paired analysis of TNF-α-stimulated versus TNF-α stimulated and ITF3756-treated monocytes, where paired student’s t-test was used for statistical analysis. **(B)** Percentage of PD-L1 positive cells and PD-L1 GMFI. For both the percentage of PD-L1 positive cells and for PD-L1 GFMI, the graphs on the left show the analysis of PD-L1 in all experimental conditions where statistical analysis was performed using RM one-way ANOVA followed by Dunnetts multiple comparison test, while the graphs on the right show the paired analysis of TNF-α-stimulated versus TNF-α stimulated and ITF3756-treated monocytes, where paired student’s t-test was used for statistical analysis. Values on the graphs are expressed as mean ± SD. *p<0,05, **p<0,001, ***p<0,0005,****p<0,0001.

After TNF−α stimulation, monocytes upregulated CD40 and PD-L1 expression (**Figure 1**). In this context, ITF3756 showed a trend towards further increasing CD40 expression levels (**Figure 1A**), while it significantly downregulated both PD-L1 expression levels and the percentage of PD-L1 positive cells (**Figure 1B**).

**Figure 2:**
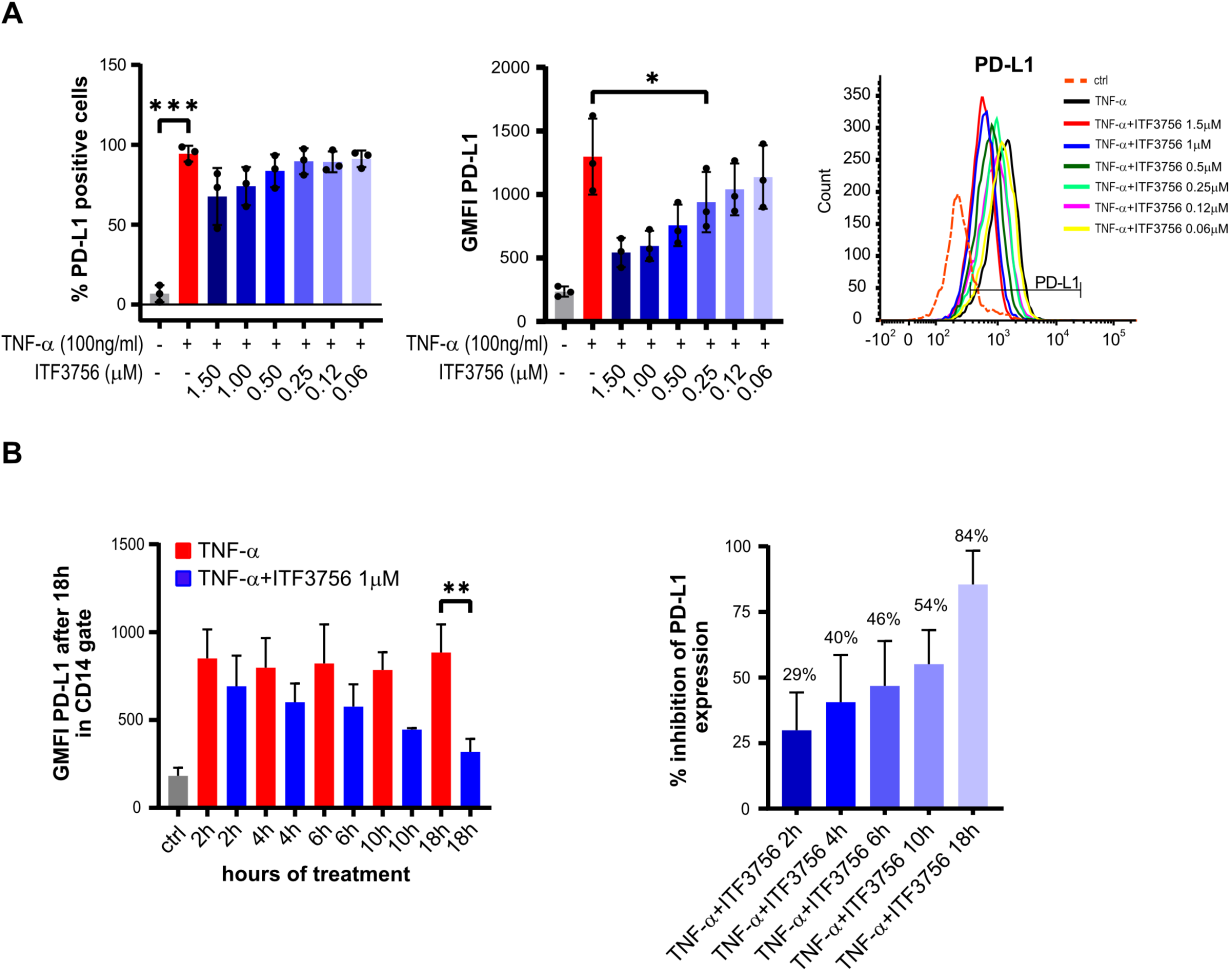
ITF3756 dose-dependently downregulates PD-L1 positive cells and expression in TNF-α stimulated monocytes and the effective PD-L1 suppression needs long exposure to the inhibitor. **(A)** Dose dependent PD-L1 inhibition by ITF3756 (1,5μM-0,0625μM). The percentage of PD-L1 positive cells and PD-L1 expression measured as the fluorescence intensity geometric mean (GMFI), together with a representative histogram of the flow cytometry results obtained, are reported. Values on the graphs represent the mean of 2 experiments carried out on 3 different donors (n=3). **(B)** Human monocytes were stimulated with TNF-α (100ng/ml) and ITF3756 1μM for 2h, 4h, 6 h, 10h and 18h. After 2h, 4h, 6 h and 10h medium was removed and replaced with fresh medium without the cytokine and ITF3756. Expression of PD-L1 was analyzed for all conditions at 18h. The left panel shows the GMFI of PD-L1 at the different time points; the right panel displays the percentage of PD-L1 inhibition at the different time points. Values on the graphs are expressed as mean ± SD. n=6 for 18h; n=4 for 2, 4 and 6h; n=2 for 10h. P-values were calculated by one-way ANOVA test followed by Dunnetts multiple comparison test. *p<0,05, **p<0,001, ***p<0,0005.

The effect of ITF3756 on PD-L1 positive cells and PD-L1 expression was dose dependent up to the highest tested dose of 1.5 µM (**Figure 2A**). Remarkably, none of the tested doses induced any cytotoxic effect, as monitored by flow cytometry analysis of 7AAD positive cells (**Supplementary Figure 1A**).

To investigate the time-dependence of PD-L1 downregulation by ITF3756, we first performed wash-out experiments treating cells with TNF-α. The data indicated that 2 hours of exposure to TNF-α are sufficient to obtain of PD-L1 upregulation, comparable to that obtained after 18 hours of continuous exposure (**Supplementary Figure 1B**). Next, wash-out experiments treating cells with both TNF-α and ITF3756 for 2h, 4h, 6h, 10h and 18h were carried out. After each time point, the medium was removed (except for the condition at 18h) and fresh medium without TNF-α and ITF3756 was added to the cells, PD-L1 expression was analyzed by flow cytometry after 18 hours for all conditions. **Figure 2B** shows that ITF3756 maximally reduced the expression level of PD-L1 when maintained in the culture for 18h. However, the percentage of PD-L1 inhibition was time-dependent and increased with the increase of ITF3756 incubation time, reaching 50% of inhibition after 10 hours of incubation (**Figure 2B**).

**Figure 3:**
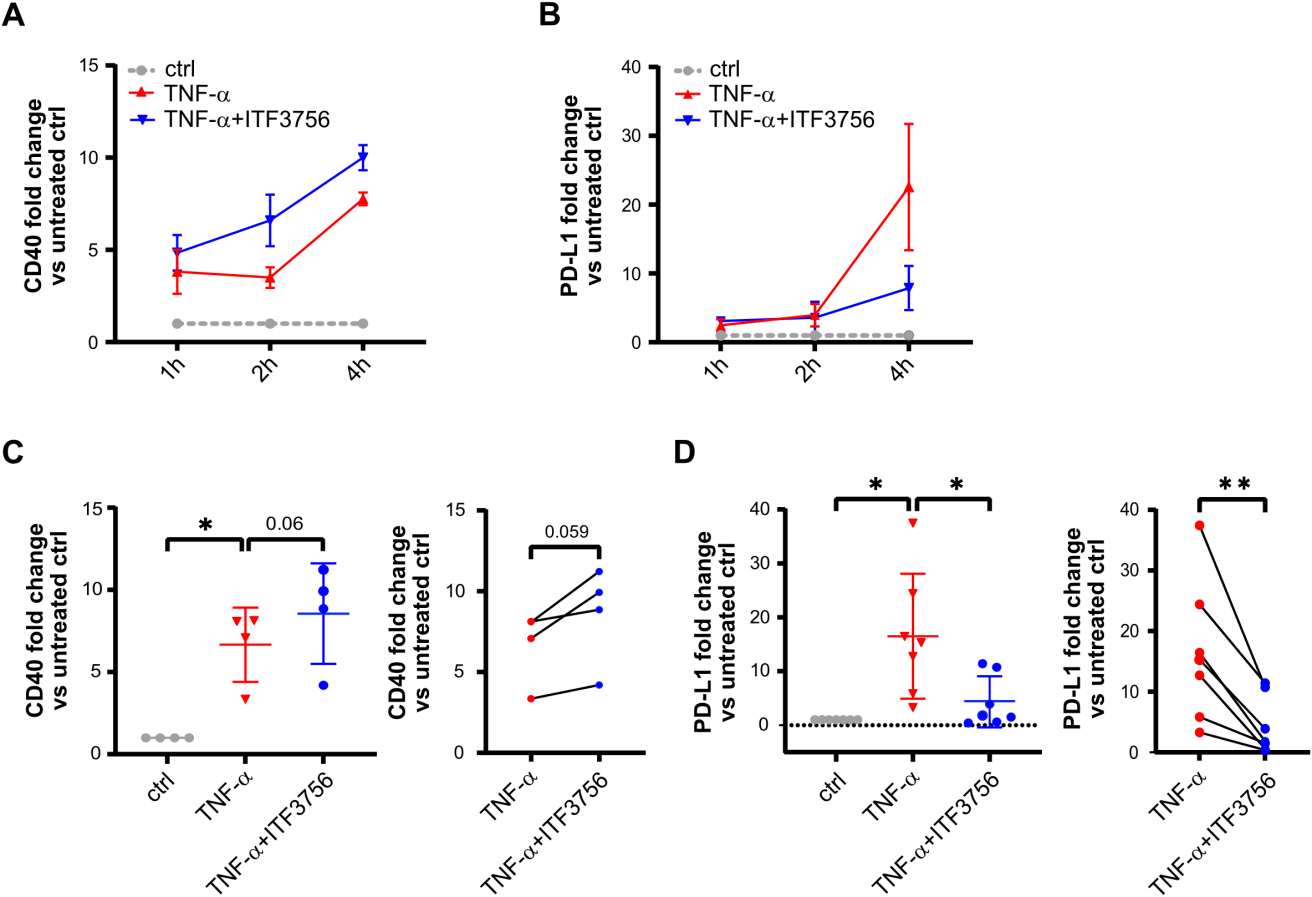
ITF3756 upregulates CD40 and decreases PD-L1 mRNA expression in TNF-α stimulated monocytes after 4h. Human purified monocytes were treated for 2h with ITF3756 (1μM) and then stimulated with TNF-α (100ng/ml) for 1, 2 and 4h. **(A)** Time course analysis of CD40 gene expression. The analysis was performed by qPCR on 3 different donors (n=3). Values on the graph are expressed as mean± SEM. **(B)** Time course analysis of PD-L1 gene expression. The analysis was performed by qPCR on 3 different donors (n=3). Values on the graph are expressed as mean± SEM. Gene expression analysis on further donors was conducted only at 4h for **(C)** CD40 (n=4) and for **(D)** PD-L1 (n=7). For **(C, D)** panel, the graph on the left shows the analysis of CD40 and PD-L1 expression in all experimental conditions in which RM one-way ANOVA test was used for the statistical analysis, while the graph on the right shows the paired analysis of TNF-α-stimulated versus TNF-α-stimulated and ITF3756-treated monocytes, in which paired student’s t-test was used for the statistical analysis. Values on the graphs are expressed as mean ± SD. *p<0,05, **p<0,001.

We next investigated whether the observed modulations of PD-L1 and CD40 occurred also at the transcriptional level. We performed a time course experiment by pre-treating monocytes for 2h with ITF3756 and then stimulating cells with TNF-α for 1h, 2h and 4h. Treatment with ITF3756 increased the expression of *CD40* starting from 1 hour up to 4h compared to TNF-α-stimulated control (**Figure 3A**), while it clearly affected *PD-L1* expression only after 4h (**Figure 3B** **).** Since we observed major changes in the gene expression of *CD40* and *PD-L1* after 4h of stimulation, we focused the analysis on this time point, increasing the number of biological replicates. As shown in **Figure 3C**, we confirmed that ITF3756 significantly decreased *PD-L1* mRNA and it slightly upregulated *CD40* expression, in TNF-α-stimulated monocytes treated for 4h (**Figures 3C, D**).

Since we aim to utilize ITF3756 as a therapeutic compound for cancer treatment, we investigated whether ITF3756 exerts the same modulatory effect on PD-L1 expression in monocytes from cancer patients as it does in monocytes collected from healthy donors. PBMC collected from one patient with colon carcinoma and one with breast cancer were treated as reported in M&M. We performed the analysis directly on PBMC instead of purified monocytes due to the small number of cells in the samples. In flow cytometry analysis, we gated the CD14 positive cells in PBMC to select monocytes population. ITF3756 decreased the percentage of positive cells and the expression of PD-L1 on monocytes in both samples (**Supplementary Figures 2A, B**).

Overall, these data suggests that HDAC6 inhibition by ITF3756 exerts a twofold effect on monocytes, being able to promote a costimulatory phenotype by inducing *CD40*, and to reduce the immunosuppressive phenotype downregulating *PD-L1* at the same time.

### ITF3756 inhibits TNF-α pathways activation and promotes a less immunosuppressive phenotype in TNF-α stimulated monocytes

ITF3756 drives TNF-α stimulated monocytes towards a less suppressive phenotype evidenced by the down-modulation of PD-L1 and the moderate upregulation of CD40. Starting from this evidence, we further investigated the effect of ITF3756 on monocytes both at mRNA and protein expression level by transcriptomic and proteomic analysis of TNF-α stimulated monocytes treated with ITF3756 for 4h and 18h respectively.

Starting from a previously published RNA sequencing dataset (34) we carried out a deeper analysis and additionally, we corroborated this data performing an extensive proteomics analysis on TNF-α -stimulated monocytes subjected to ITF3756 treatment for 18 hours.

**Figure 4:**
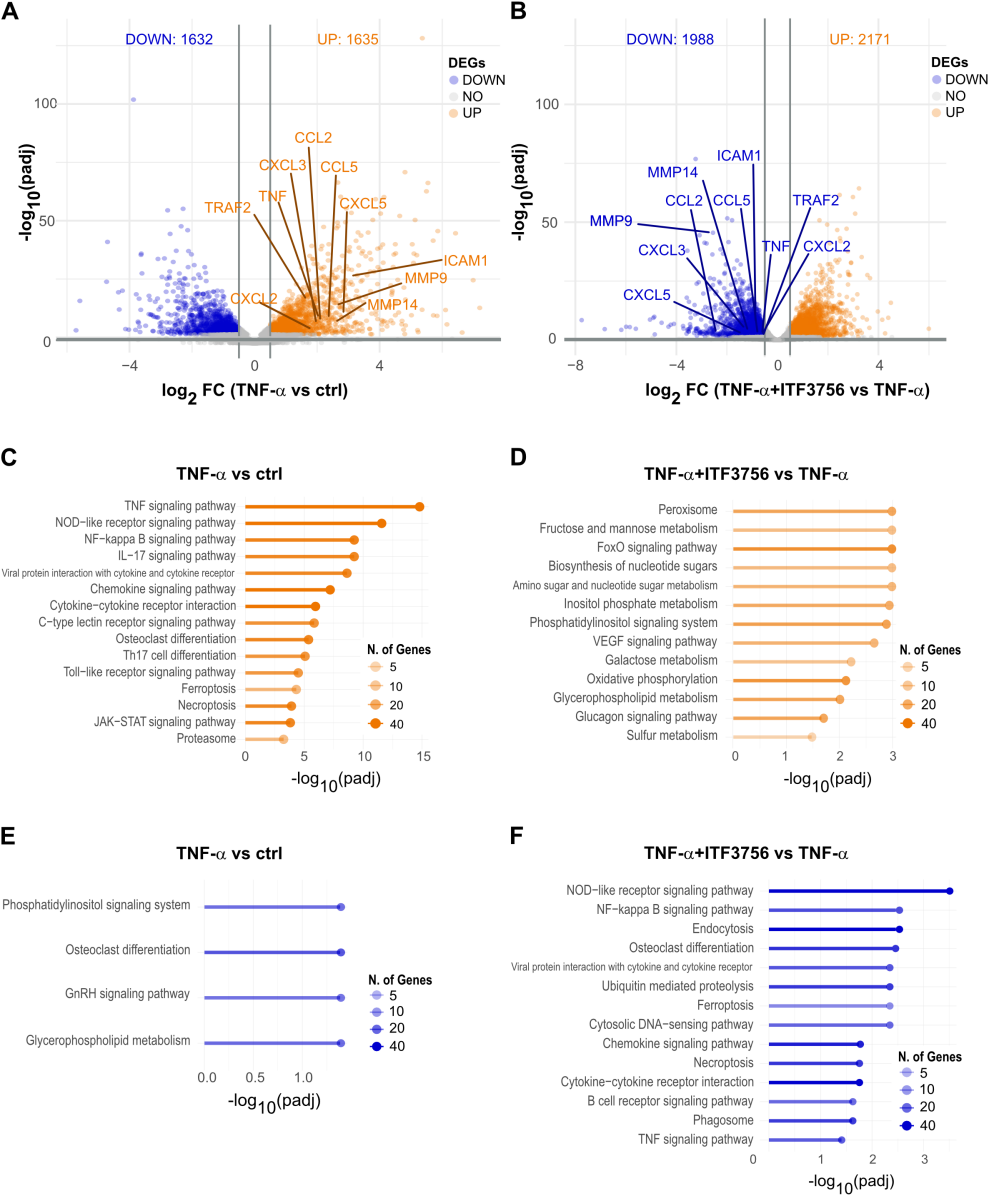
Transcriptomic analysis of TNF−α stimulated monocytes treated or not with ITF3756 for 4h. Purified human monocytes were treated for 2h with ITF3756 (1μM) and then stimulated with TNF-α (100ng/ml) for 4h. **(A)** Volcano plot displaying significantly up- and down-regulated genes (orange and blue, respectively) in monocytes stimulated with TNF-α. Fold changes (FC) are calculated versus the unstimulated control cells. **(B)** Volcano plot displaying significantly up- and down-regulated genes (orange and blue, respectively) in monocytes stimulated with TNF-α and treated with ITF3756. Fold changes (FC) are calculated versus the TNF-α; stimulated cells. **(C, E)** Pathways analyses of significantly up- and down-regulated genes (orange and blue, respectively) in TNF-α; stimulated monocytes were performed with the EnrichR software. **(D, F)** Pathways analyses on significantly up- and down-regulated genes (orange and blue, respectively) in monocytes stimulated with TNF-α; and treated with ITF3756 were performed with the EnrichR software.

Transcriptomic analysis revealed significant differential gene expression. In TNF-α-stimulated monocytes, 3267 genes were deregulated (adjusted P<0.05) (**Figure 4A**), while monocytes treated with both TNF-α and ITF3756 showed 4159 deregulated genes compared to TNF-α-stimulated cells (**Figure 4B**). In particular, the volcano plots illustrated that TNF-α treatment led to the upregulation of TNF-α pathway-related genes, including *CCL2, CXCL3, CCL5, TNF, TRAF, CXCL2*, and *ICAM-1*, while the treatment with ITF3756 resulted in the downregulation of those genes. As expected, when monocytes were stimulated with TNF-α a pathway enrichment analysis indicated strong upregulation of the TNF-α pathway (p.adj=1.59x10^-15^), as well as pathways related to monocyte activation and differentiation, including the NOD-like receptor signaling pathway, NF-kappa B signaling pathway, cytokine-cytokine receptor interaction, and JAK-STAT signaling pathway (**Figure 4C**). Conversely, downregulated genes were enriched in pathways such as the phosphatidylinositol signaling system and glycerophospholipid metabolism (**Figure 4E**).When cells were treated with both TNF-α and ITF3756, pathway enrichment analysis demonstrated upregulation of pathways related to peroxisomes and various metabolic processes, including fructose, mannose, and galactose metabolism, biosynthesis of nucleotide sugars, and oxidative phosphorylation (**Figure 4D**). In agreement with our previously published data (34), downregulated pathways included the NOD-like receptor signaling pathway, NF-kappa B signaling pathway, and TNF signaling pathway, notably enriched with several key members (**Figure 4F**, **Supplementary Figures 3A, B** **).** The upregulation of sugar metabolism and oxidative phosphorylation has been extensively described as features of activated myeloid cells after inflammatory stimuli (35, 36), thus the overrepresentation of these pathways when monocytes were treated with both compounds suggests that these cells are metabolically active even if HDAC6 inhibition determines a clear reduction of the TNF-α inflammatory pathways.

**Figure 5:**
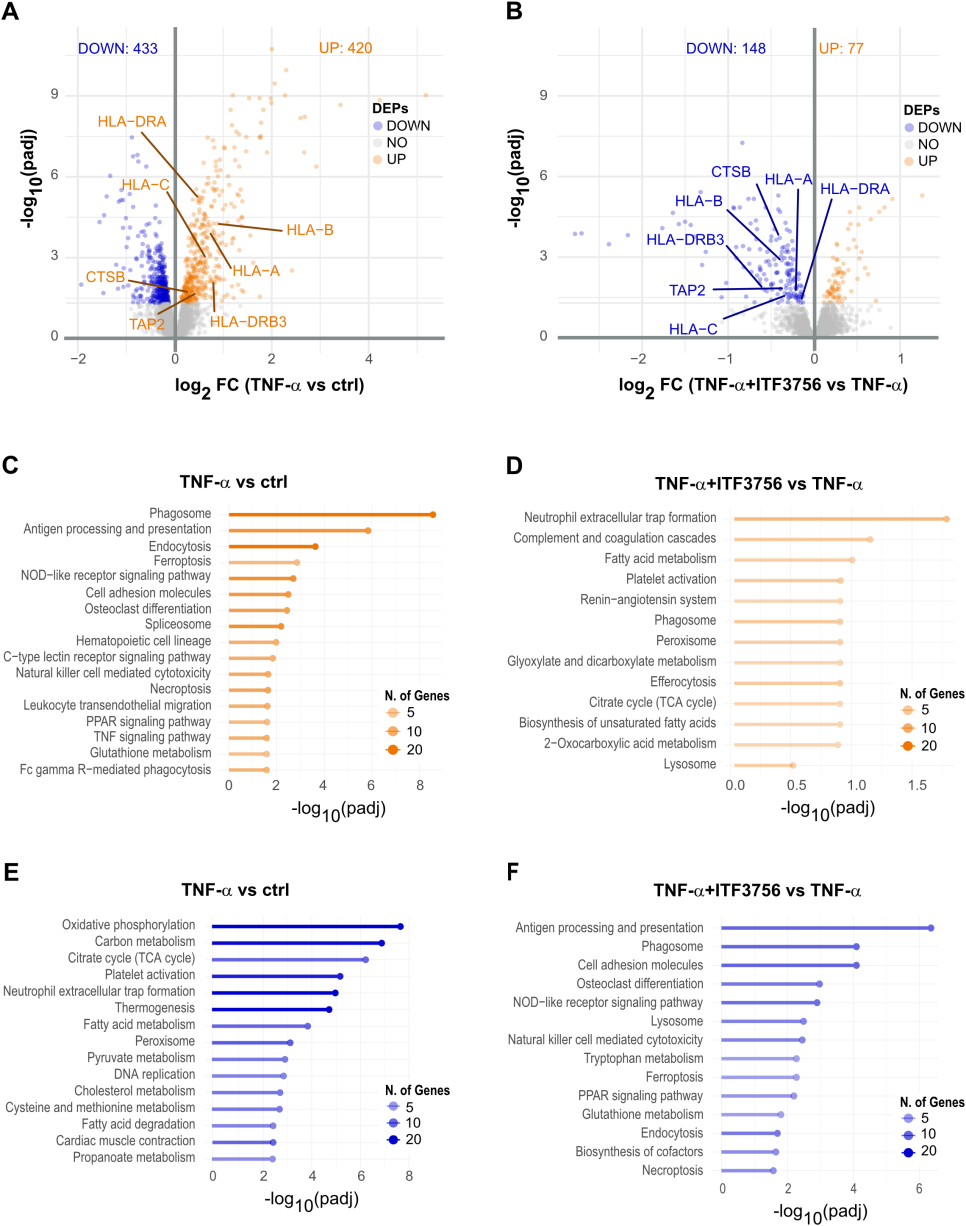
Proteomics analysis of TNF-α stimulated monocytes treated or not with ITF3756 for 18h. Purified human monocytes were treated for 2h with ITF3756 (1μM) and then stimulated with TNF-α (100ng/ml) for 18h. **(A)** Volcano plot displaying significantly up- and down-regulated proteins (orange and blue, respectively) in monocytes stimulated with TNF-α. Fold changes (FC) are calculated versus the unstimulated control cells. **(B)** Volcano plots displaying significantly up- and down-regulated proteins (orange and blue, respectively) in monocytes stimulated with TNF-α and treated with ITF3756. Fold changes (FC) are calculated versus the TNF-α stimulated cell. **(C, E)** Pathways analyses on significantly up- and down-regulated proteins (orange and blue, respectively) in TNF-α stimulated monocytes were performed with the EnrichR software. **(D, F)** Pathways analyses on significantly up- and down-regulated proteins (orange and blue, respectively) in monocytes stimulated with TNF-α and treated with ITF3756 were performed with the EnrichR software.

Proteomics analysis corroborated the transcriptomic data, showing 853 deregulated proteins in TNF-α-treated monocytes (**Figure 5A**), while in cells treated with both ITF3756 and TNF-α, 226 proteins were deregulated compared to TNF-α-treated monocytes (**Figure 5B**). Volcano plots showed antigen processing and presentation protein (HLA-DRA, HLA-C, HLA-B, HLA-AHLA-DR-B2, TAP-2 and CTSB) upregulated by TNF-α treatment and downregulated by the treatment TNF-α and ITF3756. In line with what observed at the transcripts level, pathway analysis of TNF-α-treated monocytes indicated upregulation of pathways related to monocyte activation and differentiation, such as antigen processing and presentation, PPAR signaling pathway, and TNF signaling pathway, along with non-apoptotic cell death pathways like necroptosis and ferroptosis (**Figure 5C**). Downregulated pathways in TNF-α-treated cells included oxidative phosphorylation, carbon metabolism, TCA cycle, and thermogenesis (**Figure 5E**). In contrast, cells treated with ITF3756 and TNF-α showed the upregulation of metabolic pathways such as fatty acid metabolism, TCA cycle, and 2-oxocarboxylic acid metabolism and detoxification pathways linked to phagosome, peroxisome, and lysosome activities (**Figure 5D**), and the downregulation of TNF-related pathways, including the NOD-like receptor signaling pathway, PPAR signaling pathway, ferroptosis, and necroptosis (**Figure 5F**).

Correlation analysis of mRNA and protein data was then performed. In TNF-α-treated cells 3219 species were detected at both mRNA and protein levels and among these, 368 expressed genes were significantly deregulated; notably, 338 (91.8%) exhibited concordant alterations, while 30 showed opposite deregulation patterns (**Supplementary Figure 4A**). In monocytes treated with both ITF3756 and TNF-α, 3234 mRNA and protein species were commonly detected, with 195 significantly deregulated at both RNA and protein levels. Among these, 143 (73.3%) showed concordant alterations (**Supplementary Figure 4B** **).** Pathway enrichment analysis of concordantly altered biomolecules confirmed the upregulation of the TNF signaling pathway, NOD-like receptor signaling pathway, and ferroptosis in TNF-α-treated cells, and the increase of fatty acid metabolism and peroxisome pathways in ITF3756 and TNF-α-treated monocytes (**Supplementary Figures 4C, D**). Analysis of downregulated pathways showed significant downregulation of efferocytosis and peroxisome pathways in TNF-α-treated cells, while phagosome, PPAR signaling pathway, and ferroptosis were confirmed to be downregulated in monocytes treated with both ITF3756 and TNF-α compared to those treated with TNF-α alone (**Supplementary Figures 4E, F**).

**Figure 6:**
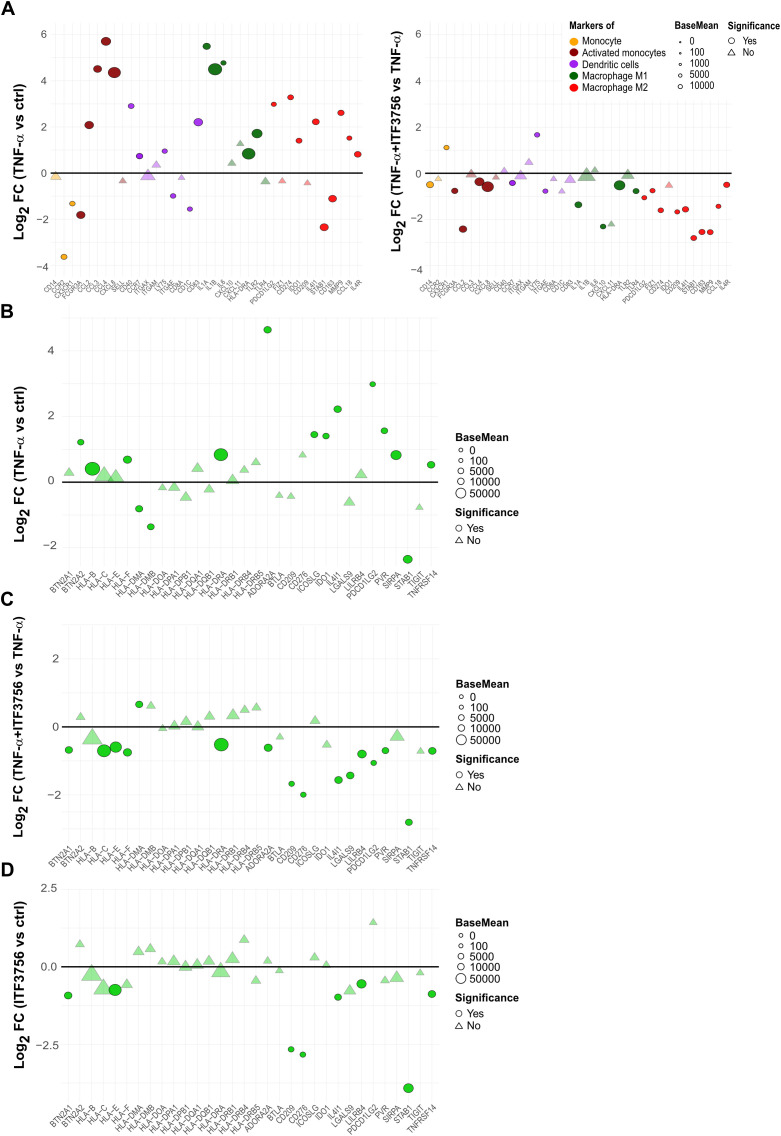
ITF3756 downregulates monocytes activation and differentiation markers activated by TNF-α and promotes a less immunosuppressive phenotype in TNF-α stimulated monocytes. Purified human monocytes were treated for 2h with ITF3756 (1μM) and then stimulated with TNF-α (100ng/ml) for 4h. RNAseq data obtained as described before were used for this analysis. **(A)** Analysis of the modulation of specific markers of monocytes-derived cell population by TNF-α (left panel) and by the combination of TNF-α and ITF3756 (right panel). Fold changes (FC) are calculated versus the unstimulated control cells or versus the TNF-α stimulated cells, respectively. **(B–D)** Analysis of the modulation by TNF-α and by the combination of TNF-α and ITF3756 of a list of inhibitory immune checkpoints (31). Fold changes (FC) are calculated versus the unstimulated control cells in **(B)**, versus the TNF-α stimulated cells in **(C)** and between ITF3756 and unstimulated control cells in **(D)**. Significant differentially expressed genes are represented as circles, while non-significant genes are shown as triangles.

Furthermore, we inspected specific markers of monocytes-derived cell population to better assess the potential effect of ITF3756 on monocytes activation or differentiation. RNAseq data showed that TNF-α significantly increased markers of activated monocytes and dendritic cells (*CCL2*, *CCL4*, *CD40* and *CD83*), M1 macrophages (*IL1B*, *HLA-DRA*, and *TLR2*), and M2 macrophages (*CD274*, *IL4I1*, *MMP9*, and *IL4R*) (**Figure 6A**, left panel). ITF3756 treatment slightly downregulated most of these markers compared to TNF-α-treated monocytes, and interestingly all the M2 macrophage markers were significantly down modulated (**Figure 6A**, right panel).

We investigated whether, besides *PD-L1*, other immune checkpoint genes were regulated by ITF3756. We thus analyzed the effect of ITF3756 on a list of genes reported in the literature as inhibitory immune checkpoints (37) in TNF-α-stimulated monocytes. TNF−α modulated the expression of these genes (**Figure 6B** **),** ITF3756 significantly downmodulated them compared to TNF-α-stimulated monocytes (**Figure 6C**). To better assess if this modulation was only due to the ability of ITF3756 to counteract TNF-α effect, we looked at the results obtained by treating monocytes with ITF3756 compared to vehicle-treated control cells (**Figure 6D**). Several of the inhibitory immune checkpoints resulted downmodulated by the HDAC6i also in vehicle treated monocytes, confirming that the drug is a transcriptional regulator of immune checkpoint molecules also in the absence of a pro-inflammatory stimulus.

### Signaling pathways involved in PD-L1 regulation by ITF3756 in TNF-α stimulated monocytes

PD-L1 expression is modulated by a complex network of regulatory mechanisms that includes genomic alteration, transcriptional regulation, post-transcriptional and post-translational modifications (38).

**Figure 7:**
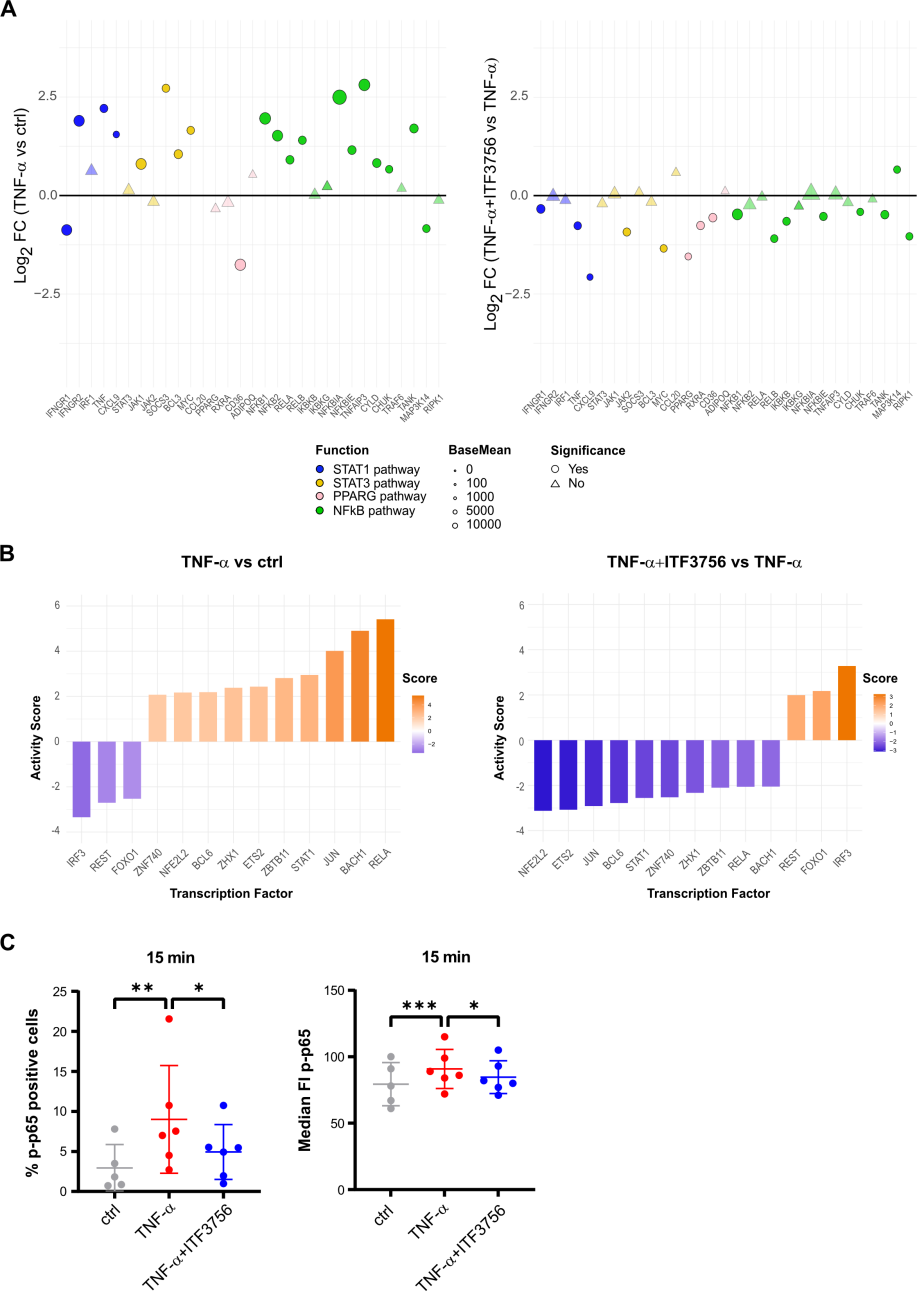
Signaling pathways involved in PD-L1 regulation by ITF3756 in TNF-α stimulated monocytes. Purified human monocytes were treated for 2h with ITF3756 (1μM) and then stimulated with TNF-α (100ng/ml). **(A)** Analysis of the modulation by TNF-α (left panel) and by the combination of TNF-α and ITF3756 (right panel) of specific pathways involved in PD-L1 regulation. RNAseq data obtained as described before were used for this analysis. Significant differentially expressed genes are represented as circles, while non-significant genes are shown as triangles. **(B)** Transcription factor activity inference upon treatment with TNF-α (left panel) and with the combination of TNF-α and ITF3756 (right panel). **(C)** Cytofluorimetric analysis of p65 phosphorylation on Ser536 (p-p65). Monocytes were pre-treated for 2h with ITF3756 and then stimulated or not with TNF-α for 15 minutes. RM one-way ANOVA followed by Dunnetts multiple comparison test was used for the statistical analysis. The graphs show the results obtained from 6 different donors. *p<0,05, **p<0,001, ***p<0,0005.

To investigate the possible mechanisms involved in *PD-L1* downregulation in monocytes treated with ITF3756, we further analyzed RNAseq data focusing on some of the pathways that are known to be involved in *PD-L1* regulation (38). Among them we looked at the NF-kappa B pathway and various JAK-STAT pathways (STAT1, STAT3). TNF-α significantly increased the expression of pathway members such as *IFNGR2*, *JAK1* (for STAT1, STAT3), and *NFKB1*, *NFKB2*, *RELA*, and *RELB* (for the NF-kappa B pathway) (**Figure 7A**). ITF3756 effectively hindered this upregulation, resulting in most pathway members being downregulated, in particular those belonging to the NF-κB signaling (**Figure 7A**; **Supplementary Figures 3A**, **5**). Analysis of transcription factor activity further confirmed these findings. In monocytes treated with TNF-α, we observed a significant activation of RELA and STAT1, key regulators of the NF-κB and JAK-STAT pathways, respectively, along with the inactivation of IRF3. Conversely, in cells treated with both TNF-α and ITF3756, we observed the opposite trend, with RELA and STAT1 being inactivated and IRF3 showing increased activity (**Figure 7B**). The lists of transcription factor target genes are provided in **Supplementary Tables 1**-**3**.

Monocyte activation, macrophage differentiation, and the infiltration of myeloid cells into the TME are complex processes regulated by multiple genes, with the JAK-STAT and NF-κB pathways playing central roles. To provide a more comprehensive overview of key gene alterations and the regulatory mechanisms involved, we analyzed the expression of genes that are direct targets of RELA and STAT1. Our findings show that the increased activity of these transcription factors in TNF-α-treated cells correlates with an upregulation of their target genes. In contrast, ITF3756 treatment reduced this activation, mirroring the observed decrease in transcription factor activity (**Supplementary Table 4**).

In addition to the transcriptional regulation, we tested whether ITF3756 could modulate *PD-L1* expression at the post-transcriptional level. Therefore, we focused on NF-kappa B pathway, and we evaluated by cytofluorimetric analysis the phosphorylation of serine 536 of the p65 subunit (p-p65) of NF-κB after 15 minutes of monocytes stimulation with TNF-α alone or in combination with ITF3756 treatment. As expected, TNF-α significantly increased the percentage of cells with phosphorylated p65, as well as the median fluorescence intensity of p-p65, while ITF3756 effectively counteracted TNF-α effect, suggesting that the HDAC6i can also regulate the activation of p65, besides regulating the expression of NF-kappa B family members (**Figure 7C**).

### ITF3756 treatment of TNF-α- activated monocytes enhances allogenic T cells proliferation

**Figure 8:**
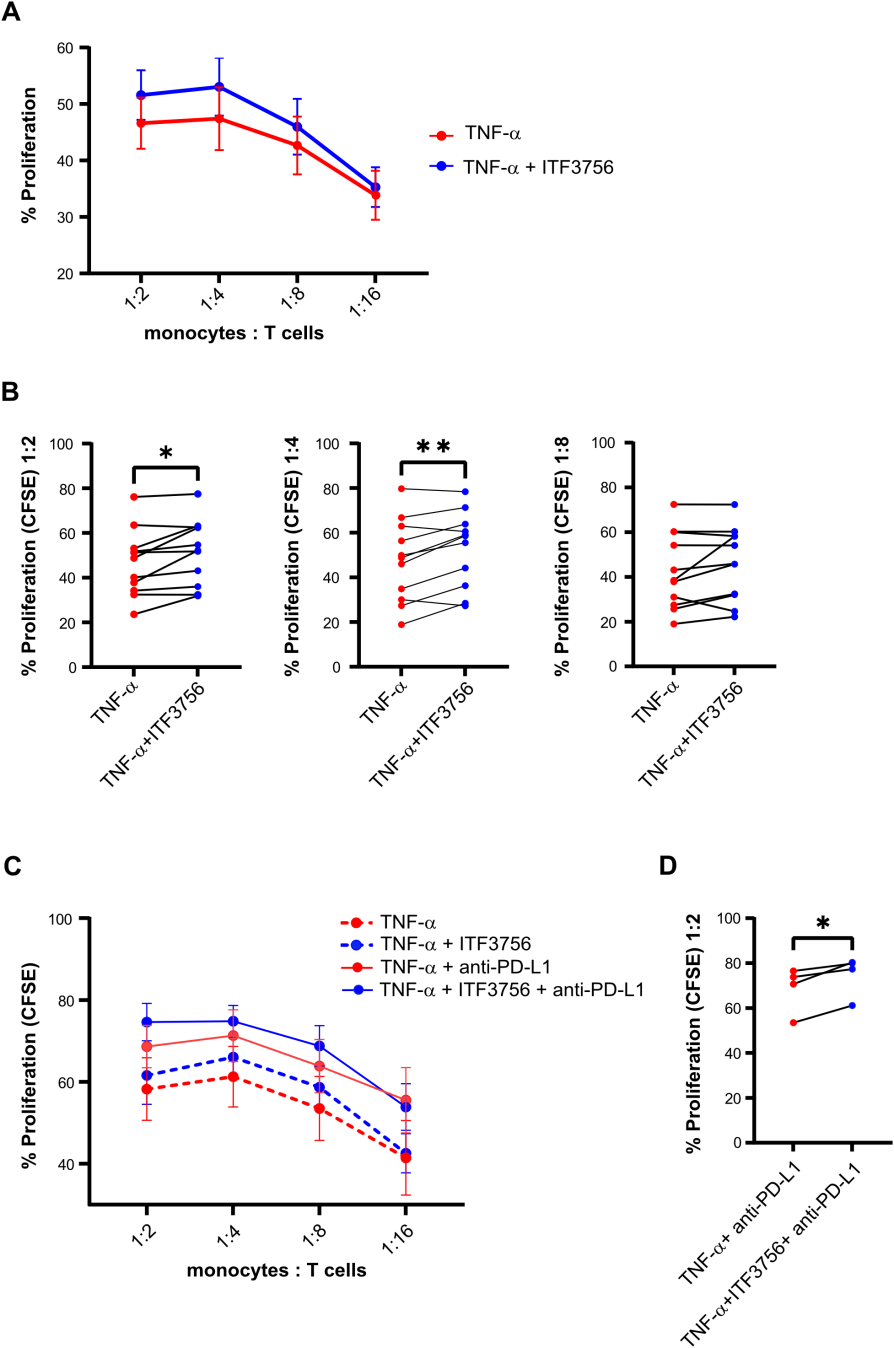
ITF3756 promotes T cells proliferation induced by TNF-α in monocytes. Human monocytes were pre-treated with ITF3756 (1μM) and stimulated with TNF-α (100ng/ml) ON. After incubation, monocytes were washed and co-cultured with allogeneic CFSE-labelled T cells at the indicated ratios. Proliferation was measured on day 6 as CFSE dilution. **(A)** Proliferation of T cells in co-culture with TNF-α-stimulated monocytes treated or not treated with ITF3756; values are expressed as mean ± SEM. Values on the graphs represent the mean of 6 experiments carried out on 12 different donors (n=12) **(B)** Paired analyses of T cell proliferation in the co-culture at 1:2, 1:4 and 1:8 monocytes/T cells ratios in TNF-α-stimulated monocytes treated or not with ITF3756. **(C)** Proliferation of T cells in co-culture with TNF-α stimulated monocytes treated or not treated with ITF3756 (dotted lines), and together with an anti-PD-L1 antibody (solid lines). Values are expressed as mean ± SEM. Values on the graphs are the mean of 2 experiments carried out on 4 different donors (n=4). **(D)** Paired analysis of T cell proliferation in the co-culture at 1:2 monocytes/T cells. P values were calculated by Student’s t-test, using a Paired t test. *p<0,05, **p<0,01.

ITF3756 significantly downmodulates PD-L1 at both protein and gene expression levels and this effect is associated with an upregulation of the costimulatory molecule CD40. In addition, our data points out that ITF3756 treated monocytes are associated with the reduction of several immune checkpoint inhibitors, beyond PD-L1. Based on these data, we hypothesized that HDAC6i treated monocytes could have a greater ability to activate T cells compared to those treated only with TNF-α. To evaluate this hypothesis, monocytes treated with ITF3756 and subsequently stimulated with TNF-α were co-cultured at different monocyte/T cell ratio with CFSE-labelled allogeneic T cells in MLR assay. T cell proliferation was quantified after 6 days of co-culture by the measurement of CFSE dilution by flow cytometry. The *in vitro* pre-treatment of human monocytes with ITF3756, followed by TNF-α activation, induced a greater alloreactive T cell proliferation compared to TNF-α treated monocytes (**Figure 8A**). In particular, to improve data interpretation, limited by the variability between the donors, we plotted and reanalyzed the data in paired graphs, separating monocyte/T cell ratio combinations. The paired analysis of samples treated or not with ITF3756 at each monocyte/T cell ratio revealed a significant increase in T cell proliferation when cells were treated with the HDAC6i (**Figure 8B**).

Moreover, since the effect of ITF3756-treated monocytes on CD3 T cells proliferation could be in part dependent on PD-L1 modulation, we investigated whether the ITF3756 treatment in combination with an anti-PD-L1 antibody could further influence T cells proliferation in an MLR assay. An anti-PD-L1 antibody was thus added to the T cells- treated-monocytes co-culture and the T cell proliferation was assessed. The anti-PD-L1 antibody increased T cell proliferation compared to the control (**Figure 8C**). In all conditions in which monocytes were pre-treated with ITF3756 we observed an increased proliferation of T cells, in particular, when ITF3756-treated monocytes were co-cultivated with T cells in presence of the anti-PD-L1 antibody (**Figure 8D**).

### ITF3756 enhances the APCs phenotype of iDCs and mDCs, strengthening the allogeneic T cell proliferation

Given that activated monocytes treated with ITF3756 exhibited a phenotype indicative of enhanced co-stimulatory activity, as evidenced by increased T cell proliferation, we evaluated whether ITF3756 has a similar effect on other myeloid cells, such as DCs, that are the antigen-presenting cells (APCs) crucial for the regulation of the adaptive immune response.

Purified monocytes were differentiated into immature dendritic cells (iDCs) in the presence of ITF3756 (1-0.5μM), and then iDC were activated with LPS to induce maturation. On both DC types the expression of the co-stimulatory molecule CD86 and of PD-L1 was analyzed at the end of the differentiation or activation period.

**Figure 9:**
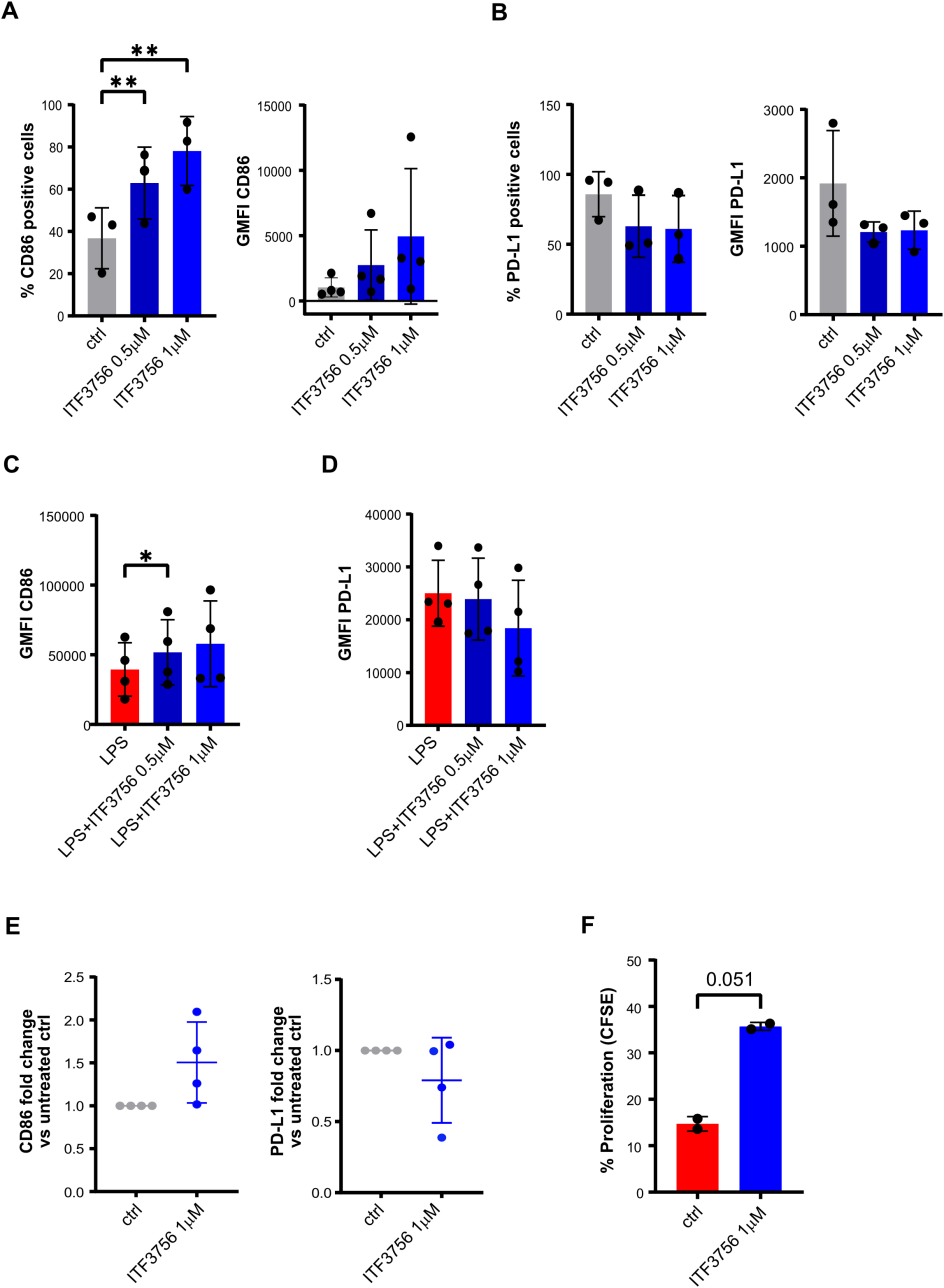
ITF3756 enhances the APCs phenotype of immature and mature dendritic cells supporting allogenic T cells proliferation. Human monocytes were treated with ITF3756 (1μM and 0,5μM) and stimulated with GMCSF/IL-4 for 5 days. After incubation, iDC were collected and assessed by flow cytometer and by qPCR. **(A)** Percentage of CD86 positive cells and CD86 GMFI. **(B)** Percentage of PD-L1 positive cells and PD-L1 GMFI. **(C, D)** Differentiated immature dendritic cells were treated with ITF3756 (1μM and 0,5μM) and stimulated with LPS (1μg/ml) for 18h. After incubation mDC cells were collected and evaluated by flow cytometry. **(C)** Expression of CD86 in mDC reported as the GMFI. **(D)** Expression of PD-L1 in mDC reported as the GMFI. **(E)** Immature dendritic cells, differentiated in the presence of ITF3756 (1μM), were collected and mRNA extracted for CD86 and PD-L1 gene expression analysis by qPCR. Values on the graphs **(A–E)** represent the mean of 2 separate experiments carried out on at least 3 different donors. **(F)** Dendritic cells differentiated in the presence of ITF3756 1μM for 5 days, were collected and co-cultured with CFSE-labelled T cells (DC/T cells ratio 1:10) for 5 days. Proliferation was measured by flow cytometry on day 5 as CFSE dilution. Values on the graph represent the mean of 2 different donors (n=2).

ITF3756 increased the percentage of positive cells and the surface expression of CD86 in iDCs differentiated in presence of ITF3756 at both concentrations used (**Figure 9A**). In iDCs, ITF3756 slightly decreased PD-L1 percentage and surface expression equally at 1 and 0,5μM (**Figure 9B**). Moreover, ITF3756 induced the expression of CD86 in mDCs (**Figure 9C**), but not the percentage of CD86-positive cells, since, as expected, after the activation with LPS, 100% of mDCs were positive for CD86 in all the conditions tested (data not shown). In mDCs, ITF3756 decreased PD-L1 expression (**Figure 9D**), but no differences in the percentage of positive cells were observed, since as well as for CD86, 100% of the cells were PD-L1 positive after LPS activation (data not shown). Gene expression analysis was in line with protein expression analysis. Indeed, treatment with ITF3756 increased *CD86* gene expression and slightly decreased *PD-L1* (**Figure 9E**). The data obtained from different donors, showed that ITF3756 clearly upregulated CD86 in both iDCs and mDCs, while PD-L1 modulation was more modest than in monocytes. Next, we performed an MLR assay by co-culturing monocyte-derived iDCs with CFSE-labelled allogeneic T cells. iDCs differentiated in presence of ITF3756 were able to enhance T cells proliferation compared to untreated control iDCs, in agreement with the data obtained with ITF3756 treated monocytes (**Figure 9F**) and confirming that the features acquired by DCs after HDAC6 inhibition improve their capacity to boost T cell proliferation.

### ITF3756 is effective *in vivo* in CT26 colon carcinoma mouse model

We investigated the effect of ITF3756 in an *in vivo* model, performing a dose response study to evaluate the efficacy of ITF3756 in controlling tumor growth. We used a model of CT26 murine colon carcinoma, this model has been used in cancer research as preclinical tumor model for immunotherapeutic drug discovery. We tested ITF3756 at 2 different doses (25 mg/kg and 50 mg/kg) with 3 different schedules of administration (once a day (QD), twice a day (BID), three times a day (TID)).

**Figure 10:**
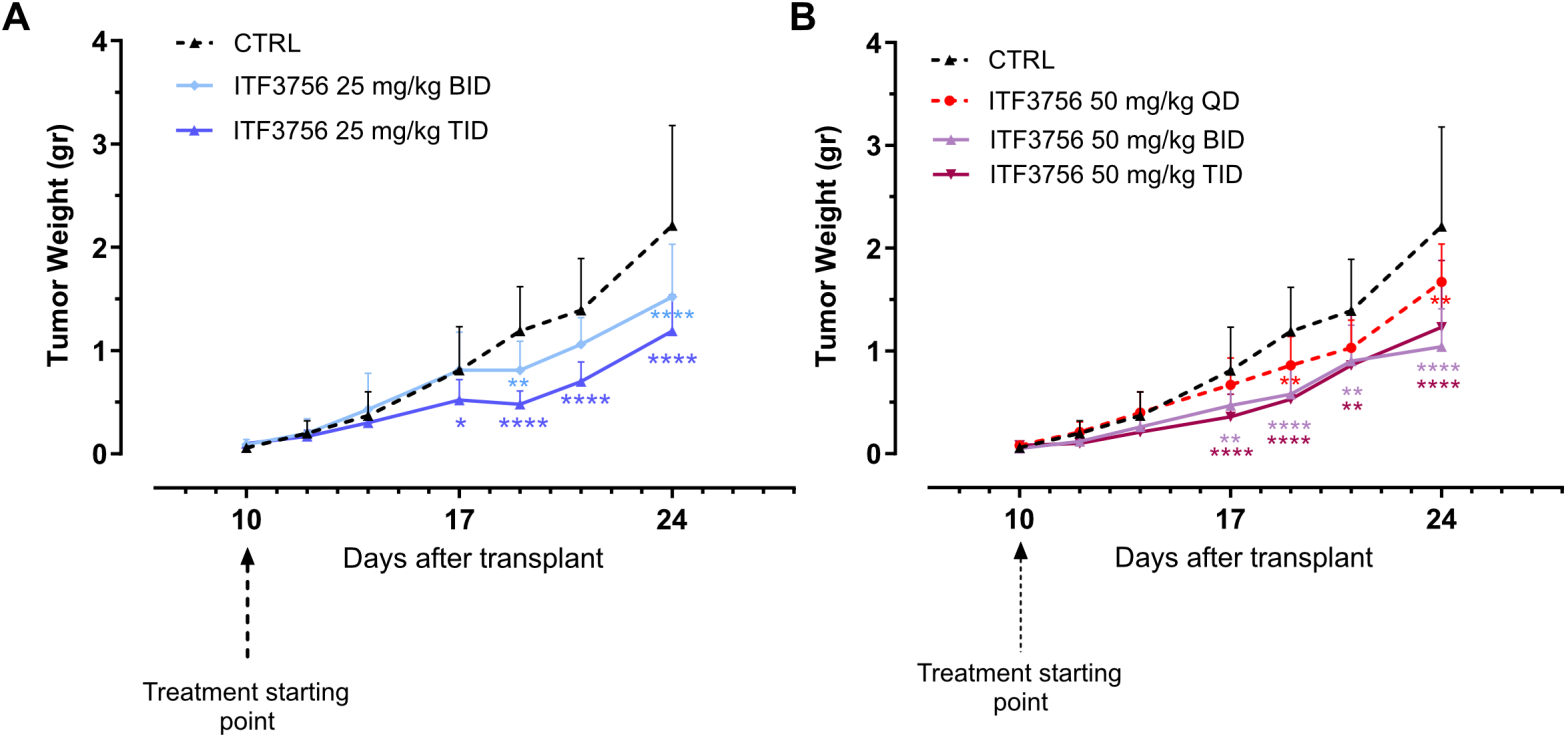
ITF3756 is effective in a colon carcinoma model *in vivo*. **(A)** Tumor weight in animals treated with ITF3756 25mg/kg BID and TID. **(B)** Tumor weight in animals treated with ITF3756 50mg/kg QD, BID and TID. Data are reported as mean ± SEM. Statistical analysis was carried out by two-way ANOVA followed by Tukey’s multiple comparisons test. *p<0,05, **p<0,001, ***p<0,0005, ****p<0,0001.

At the dose 25 mg/Kg, animals treated with ITF3756 TID showed a significant reduction of tumor growth compared to vehicle group, starting from day 17 up to the end of the experiment (46% inhibition), instead treatment BID displayed a weaker effect in controlling tumor growth, although a significant inhibition was observed at day 19 and 24 (maximum effect of inhibition, 31%) (**Figure 10A**). ITF3756 at 50 mg/Kg, when administered BID or TID, reduced significantly the tumor growth starting from day 17 throughout all the study, with maximum effect of inhibition, 55% for TID and 52% for BID, respectively (**Figure 10B**). Mice treated QD showed a significant reduction of tumor at day 19 and 24 with maximum of inhibition 27% (**Figure 10B**). Overall, 50 mg/kg ITF3756 TID and BID were the most efficacious doses. The *in vivo* data demonstrated an anti-tumoral effect of ITF3756 in a murine model of colon carcinoma and suggest that frequent administration of ITF3756 is necessary to maintain highest anti-tumor efficacy.

## Discussion

Epigenetics plays a crucial role in regulating immune cell activation, differentiation, and the overall immunological response in the whole body and within the TME (39). It not only influences tumor immunogenicity but also shapes the activity of immune cells involved in antitumor defense. Thus, it has become increasingly clear that epigenetic drugs could be useful tools for cancer treatment, particularly in combination with cancer immunotherapy. Over recent years, immune checkpoint blockade has emerged as one of the most promising strategies against cancer, however its success depends on the degree of immunological tolerance developed by the tumor and consequently on the capacity to reverse it (40). One of the mechanisms that the tumor exploits to avoid immunoediting from immune surveillance is the overexpression of PD-L1 on cancer cells, leading to an elevated and uncontrolled PD-1/PD-L1 inhibitory signaling. In this context, HDAC6 selective inhibitors have proven efficacy in downregulating PD-L1 expression both on certain types of tumor cells and myeloid cells, such as DCs and macrophages (8, 16, 17, 19). Moreover, HDAC6 plays a pivotal role in controlling the inflammatory environment in the TME. For all these reasons, the idea of using HDAC6 selective inhibitors in combination with anti-PD-1/PD-L1 to improve the immune checkpoint blockade therapy has recently taken hold.

In this study, we assessed the immunomodulatory effect of our selective HDAC6i ITF3756 on monocytes, considering the role of host myeloid cells in the control of cancer progression by immune checkpoint blockade therapies (5, 6). To mimic the effect of a proinflammatory cytokine released in the TME, we stimulated purified human monocytes with TNF-α *in vitro* after ITF3756 treatment. Our data show that HDAC6 inhibition has a dual effect on monocytes: from one side it reduces monocytes immunosuppressive phenotype through the downregulation of PD-L1 expression in a dose-dependent manner, even at low concentrations; from the other, it promotes the expression of the co-stimulatory receptor CD40.

We had already demonstrated that ITF3756 lessens TNF-α and NF-kB signaling pathway in TNF-α stimulated monocyte (34). Here we confirmed the findings at both transcriptional and protein level, as resulted from the global transcriptomic and proteomic analyses. We have already reported that monocytes treated with TNF-α in the presence of ITF3756 show a gene expression profile similar to unstimulated monocytes, which are in a resting condition and therefore have a minimal pro-inflammatory phenotype, downmodulating genes related to cytokine activity and pathways, inflammatory response and Pattern Recognition Receptors (34). These data are here validated with further analysis at the transcriptional level, and supported by the proteomic analysis, that shows the down-modulation of NOD like receptor signaling and the reduction of necroptosis and ferroptosis, which are inflammation-driven types of cell death. Furthermore, here we demonstrate that ITF3756 not only impinges on the transcription of components of the NF-kB pathway, but that it also regulates the activation of p65 reducing its phosphorylation state. Indeed, different signaling pathways are reported to be involved in the regulation of PD-L1 expression in cancer and immune cells, such as that of NF-kB, STAT1 and STAT3 (38). ITF3756 significantly dampens all of these in activated monocytes and this effect might explain the downmodulation of PD-L1 expression.

Moreover, monocytes stimulated with TNF-α and then treated with ITF3756 display a better cell fitness as suggested by the reduction of TNF-α−induced cell death pathways (41, 42) and by the overrepresentation of cell metabolic pathways. In particular, ITF3756 upregulates metabolic pathways associated with glycolysis and sugar metabolism, which are known to be increased in the initial phase of monocytes activation after LPS stimulation and enhanced in pro-inflammatory macrophages after IFNγ stimulation (43, 44).

ITF3756 downregulates several markers of M2 macrophages (e.g. *CD163* and *CD209*) compared to TNF-α stimulated monocytes. Although macrophages represent a heterogeneous cell population, they are usually classified into pro-inflammatory M1-like and anti-inflammatory M2-like showing *in vivo* a wide range of different phenotypes in between depending on the microenvironment. Indeed, TAM can continuously shift between these two phenotypes due to their inherent plasticity in response to TME signals (45). However, M2-like TAMs are the prevalent subtype in the TME, where they can promote tumor initiation, growth, metastasis and drug resistance (45). Moreover, M2 macrophages dampen the anti-cancer effect of cytotoxic T cells and support the recruitment of immunosuppressive T regulatory cells to the TME, thus fostering an immunosuppressive environment. In this regard, ITF3756 downregulated the expression of *CCL2* and *CXCL8*, which are two chemokines related to monocytes activation but also reported to be secreted by tumor infiltrating monocytes and TAMs, and promoting tumor growth and an immunosuppressive tumor environment, directly affecting tumor cells or recruiting pro-tumorigenic immune cells in TME (46–48). Thus, given their role in promoting tumor progression, the development of therapeutic strategies targeting M2-like TAMs or regulating M1/M2 polarization has become a significant area of cancer therapy research. Our observation of ITF3756 downregulating M2 macrophage markers is particularly interesting, and in line with other studies describing HDAC6 inhibitors as modulators of macrophage polarization in favor of a less anti-inflammatory phenotype (49, 50).

Together with the effect of ITF3756 on M2 markers, we also assessed the effect of our inhibitor on other immune checkpoint genes, and we found that, besides PD-L1, ITF3756 downregulates several inhibitory immune checkpoints. This occurred when monocytes were stimulated with TNF-α, and even when unstimulated monocytes were treated with only the inhibitor, confirming that this effect is independent from the pro-inflammatory stimulus. Among the regulated immune checkpoints, interleukin-4 induced 1 (IL4I1) is lately gaining interest as a novel metabolic immune checkpoint that makes the TME more immunosuppressive, thereby increasing the resistance to anti-PD-L1 therapies (51, 51).Targeting IL4I1, or reducing its expression, could be therefore an interesting therapeutic opportunity, especially in combination with PD-L1 reduction. Another checkpoint downregulated by ITF3756 is Stabilin-1 (STAB1), also known as Clever-1. STAB1 is a scavenger and adhesion receptor expressed in human monocytes, in M2-macrophages and lymphatic endothelial cells, and is involved in receptor-mediated endocytosis, angiogenesis and cell adhesion (52–55). Recently, the results from a phase I/II clinical trial show that STAB-1 blockade through the monoclonal antibody bexmarilimab leads to TAM reprogramming towards a pro-inflammatory phenotype, immune activation and tumor control in patients with late-stage cancer (56). ITF3756 also affect the gene expression level of *HLA-E* (the non-classical human leukocyte antigen-E), an antigen constitutively expressed on nucleated cells at low levels but reported to be overexpressed in multiple solid tumors and associated with worse clinical outcome in different cancers (57). High expression of HLA-E by tumor cells, TAM and dendritic cells can dampen the anti-tumor immune response of tumor-infiltrating CD8 T cells and NK cells, activating the inhibitory axis NKG2A/HLA-E (57). -Some preclinical studies have already shown that blocking HLA-E through antibodies (58, 59) or downregulating HLA-E expression through the approved anti-cancer drugs selinexor and bortezomib (60, 61) enhance NK cell cytotoxic activity in solid tumor. In our study, ITF3756 also downregulates the leukocyte immunoglobulin-like receptor 4 (*LILRB4*). This inhibitory receptor is primarily expressed on lymphoid and myeloid cells and is a component of a family that comprises 11 members that are involved in the regulation of the immune system (62, 63). Notably, LILRBs act as myeloid checkpoint receptors that restrain overt immune responses and, recently, LILRBs have been implicated in tumor progression and unfavorable therapeutic responses and therefore have been proposed as potential therapeutic targets in cancer (63, 64). In particular, in solid tumors the blockade of LILRB4 with antibodies increases infiltrating anti-tumor immune cells and decreases the inhibitory immunosuppressive cells (65).

The simultaneous modulation of multiple immune checkpoint molecules observed in monocytes treated with ITF3756 may contribute to its antitumor efficacy. Although this effect appears promising, we cannot exclude the possibility of compensatory mechanisms within the tumor microenvironment that could limit its effect. For example, Huang et al. showed that in a murine model of ovarian cancer, single-agent treatments with anti-PD-1 or anti-LAG-3 antibodies led to compensatory upregulation of other checkpoint molecules such as LAG-3 and CTLA-4, ultimately reducing therapeutic efficacy. Only combinatorial blockade (e.g., PD-1/LAG-3 or PD-1/CTLA-4) significantly improved survival (66). Unlike antibody-based approaches that fully block individual checkpoint pathways, ITF3756 induces a broader modulation of multiple immune checkpoints, which may counteract the compensatory response. Supporting this hypothesis, ITF3756 treatment significantly reduced tumor growth in a CT26 colon carcinoma mouse model. Nonetheless, further studies are required to elucidate the relationship between its immunomodulatory effects and antitumor activity *in vivo*.

Thus, here we show for the first time that ITF3756 can simultaneously downmodulate several inhibitory immune checkpoints in human monocytes, and that this occurs both in the presence and in the absence of a pro-inflammatory stimulus. The possibility of acting with a single molecule, on multiple immune checkpoints might represent a promising therapeutic opportunity, especially considering also the concomitant effect that ITF3756 seems to have on M2-macrophage reprogramming and on the CD40 costimulatory receptor expression. Moreover, these less immunosuppressive features of treated monocytes are associated with an enhancement of activatory function of monocytes. In fact, in the MLR assay, TNFα-activated monocytes treated with ITF3756 enhance allogenic T cells proliferation compared to untreated monocytes, even, and interestingly, when an anti-PD-L1 antibody is added to the co-culture. ITF3756 treatment boosts the proliferation of allogenic T cells also when differentiated DC were used in the assay, pointing up a widespread capacity of our HDAC6 inhibitor to modulate myeloid cells to stimulate T cells proliferation. The *in vivo* data confirm the potential antitumor activity of ITF3756.

Overall, our data support the use of ITF3756 in combination with immune checkpoint inhibitors in cancer therapy. Indeed, in monocytes stimulated with one of the cytokines present in the TME, TNF-α, ITF3756 is not only able to reduce one of the main targets of cancer immunotherapy, PD-L1, but it is also able to drive monocytes towards a less immunosuppressive phenotype, with reduced expression of M2 phenotypic markers, and with an increased glycolytic metabolism and with an improved capacity of inducing T cells proliferation *in vitro*. Currently, the majority of the immune checkpoint inhibitors employed in the clinics are monoclonal antibodies (mAbs). Although their proven efficacy in several tumors, they also possess relevant limitations such as the development of immune-related adverse effects (irAEs) (67). The extended half-life of mAbs and the sustained target inhibition may influence the onset of irAEs following mAbs treatment (68, 69). On the contrary, small molecules offer several advantages over antibodies, including reduced, or lack of, immunogenicity, better tissue and tumor penetration, lower production costs, and greater flexibility in optimizing pharmacokinetics. Additionally, they enable more adaptable dosing strategies to prevent immune-related adverse events (irAEs) (70, 71). In this regard, although the anti-inflammatory effect of ITF3756 on the TNF-α signaling pathway could be seen as a disadvantage for certain aspects of cancer therapy, in the context of immune checkpoint therapy combinations it could be helpful and relevant. In fact, recent studies suggest that the use of anti-TNF-α antibodies in combination with anti-immune checkpoint inhibitors reduce the adverse events and increase the antitumor immune response. It is reported that the activation of the TNF-α signaling pathway in TME, induced by a PD-1 antibody, reduces the tumor infiltrating CD8 T cells and the immune response, while blockers of TNF-α or TNFR1 (TNF-α receptor) revert this situation, decreasing CD8 T cells exhaustion and death and synergizing with anti-PD-1 (72, 73). Moreover, Perez-Ruiz et al. published that TNF-α blockers not only ameliorate the anti-tumor effect of anti-PD-1 and anti-CTLA-4 combination, but at the same time, reduce irAEs in a preclinical mouse model of colon cancer (74). However, even if the data from preclinical studies support the use of TNF-α blockers in combination with the immune checkpoint inhibitors, the clinical effect of this treatment on patients is still debated (75, 76)

In conclusion, we have reported here numerous modulations occurring in human monocytes upon HDAC6 inhibition *in vitro*. All these modulations suggest that the inhibition of HDAC6 via ITF3756 may provide a significant advantage as a cancer therapeutic approach by reducing the immunosuppressive effects of immune checkpoint molecules of myeloid cells while minimizing the toxicity typically associated with antibody-based therapies.

## Data Availability

The complete RNAseq data in this article are not readily available to preserve the intellectual propriety of the authors' company on the genes not disclosed in the present article due to a pending patent application. The proteomic data are deposited to the ProteomeXchange Consortium via the PRIDE partner repository with the dataset identifiers PXD057931.
