# Correction: HDAC6 inhibition by ITF3756 modulates PD-L1 expression and monocyte phenotype: insights for a promising immune checkpoint blockade co-treatment therapy

**DOI:** 10.3389/fimmu.2025.1645773

**Published:** 2025-08-05

**Authors:** Valeria Spadotto, Chiara Ripamonti, Andrea Ghiroldi, Elisabetta Galbiati, Pietro Pozzi, Roberta Noberini, Tiziana Bonaldi, Christian Steinkühler, Gianluca Fossati

**Affiliations:** ^1^ New Drug Incubator Department, Italfarmaco Group, Milan, Italy; ^2^ Preclinical Drug Development Department, Italfarmaco Group, Milan, Italy; ^3^ Department of Experimental Oncology, IEO European Institute of Oncology IRCCS, Milan, Italy; ^4^ Department of Oncology and Hematology-Oncology (DIPO), University of Milan, Milan, Italy

**Keywords:** HDAC6, monocytes, immuno-checkpoints, TNF-α, dendritic cells

In the published article, there was an error in **Figure 6** as published. There was a mistake in the axes of **Figure 6C** and **Figure 6D** as published, and as a result, the list of the genes were not correct. The corrected **Figure 6** and its caption “ITF3756 downregulates monocytes activation and differentiation markers activated by TNF-α and promotes a less immunosuppressive phenotype in TNF-α stimulated monocytes. Purified human monocytes were treated for 2h with ITF3756 (1μM) and then stimulated with TNF-α (100ng/ml) for 4h. RNAseq data obtained as described before were used for this analysis. **(A)** Analysis of the modulation of specific markers of monocytes-derived cell population by TNF-α (left panel) and by the combination of TNF-α and ITF3756 (right panel). Fold changes (FC) are calculated versus the unstimulated control cells or versus the TNF-α stimulated cells, respectively. **(B–D)** Analysis of the modulation by TNF-α and by the combination of TNF-α and ITF3756 of a list of inhibitory immune checkpoints (31). Fold changes (FC) are calculated versus the unstimulated control cells in **(B)**, versus the TNF-α stimulated cells in **(C)** and between ITF3756 and unstimulated control cells in **(D)**. Significant differentially expressed genes are represented as circles, while non-significant genes are shown as triangles” appear below.

**Figure 6:**
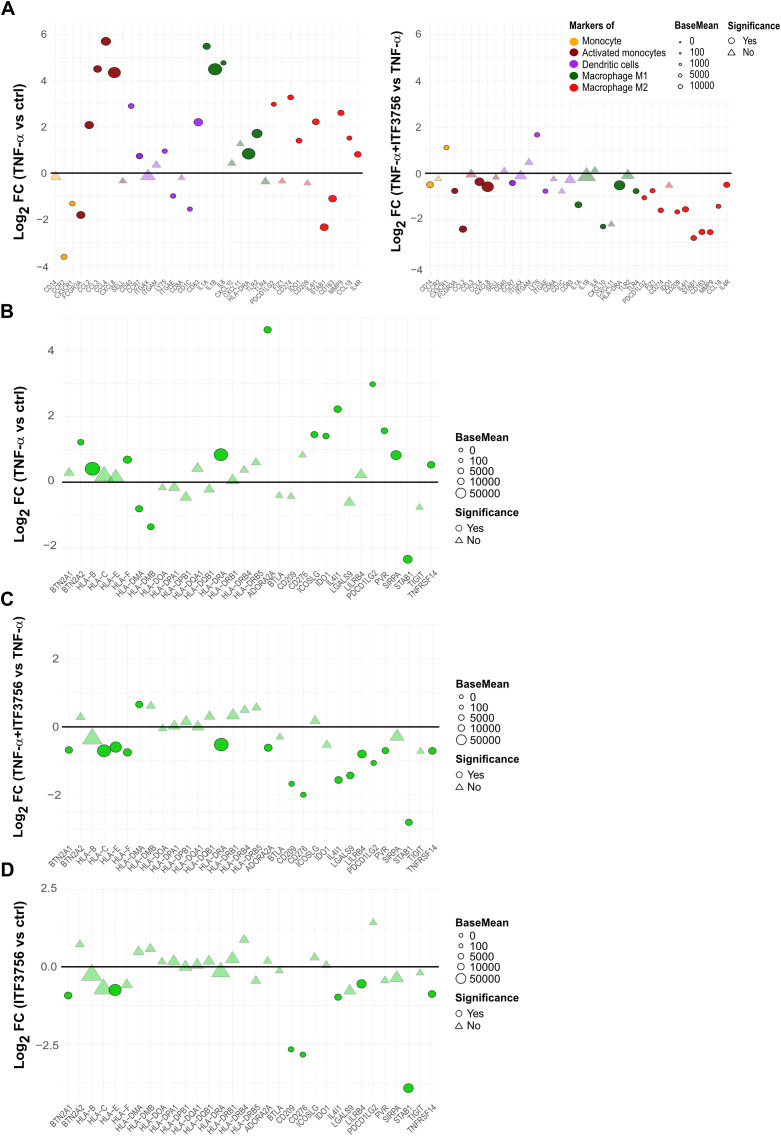
ITF3756 downregulates monocytes activation and differentiation markers activated by TNF-α and promotes a less immunosuppressive phenotype in TNF-α stimulated monocytes. Purified human monocytes were treated for 2h with ITF3756 (1μM) and then stimulated with TNF-α (100ng/ml) for 4h. RNAseq data obtained as described before were used for this analysis. **(A)** Analysis of the modulation of specific markers of monocytes-derived cell population by TNF-α (left panel) and by the combination of TNF-α and ITF3756 (right panel). Fold changes (FC) are calculated versus the unstimulated control cells or versus the TNF-α stimulated cells, respectively. **(B–D)** Analysis of the modulation by TNF-α and by the combination of TNF-α and ITF3756 of a list of inhibitory immune checkpoints (31). Fold changes (FC) are calculated versus the unstimulated control cells in **(B)**, versus the TNF-α stimulated cells in **(C)** and between ITF3756 and unstimulated control cells in **(D)**. Significant differentially expressed genes are represented as circles, while non-significant genes are shown as triangles.

The original article has been updated.

