# Supplementary material for: HDAC6 inhibition by ITF3756 modulates PD-L1 expression and monocyte phenotype: insights for a promising immune checkpoint blockade co-treatment therapy
Source: Front Immunol. 2025 May 13;16:1546939. doi: 10.3389/fimmu.2025.1546939 (PMC12106391; doi:10.3389/fimmu.2025.1546939)
Supplement: Supplementary file 1 [file DataSheet1.docx]

*Supplementary Material*

HDAC6 Inhibition by ITF3756 Modulates PD-L1 Expression and Monocyte Phenotype: Insights for a Promising Immune Checkpoint Blockade co-treatment therapy

Valeria Spadotto^1†^, Chiara Ripamonti^1†^, Andrea Ghiroldi^1^, Elisabetta Galbiati^2^, Pietro Pozzi^2^, Roberta Noberini^3^, Tiziana Bonaldi^3,4^, Christian Steinkuhler^1^ and Gianluca Fossati^1*^

^1^ New Drug Incubator Department, Italfarmaco Group, Cinisello Balsamo (Milan), Italy

^2^ Preclinical Drug Development Department, Italfarmaco Group, Cinisello Balsamo (Milan), Italy

^3^ Department of Experimental Oncology, IEO European Institute of Oncology IRCCS, Milan, Italy

^4^ Department of Oncology and Hematology-Oncology (DIPO), University of Milan, Milan, Italy

^†^These authors have contributed equally to this work and share first authorship

**Content:**

- **Supplementary Figure 1;**
- **Supplementary Figure 2;**
- **Supplementary Figure 3;**
- **Supplementary Figure 4;**
- **Supplementary Figure 5;**
- **Supplementary Table 1;**
- **Supplementary Table 2;**
- **Supplementary Table 3.**

**Supplementary Figure 1**
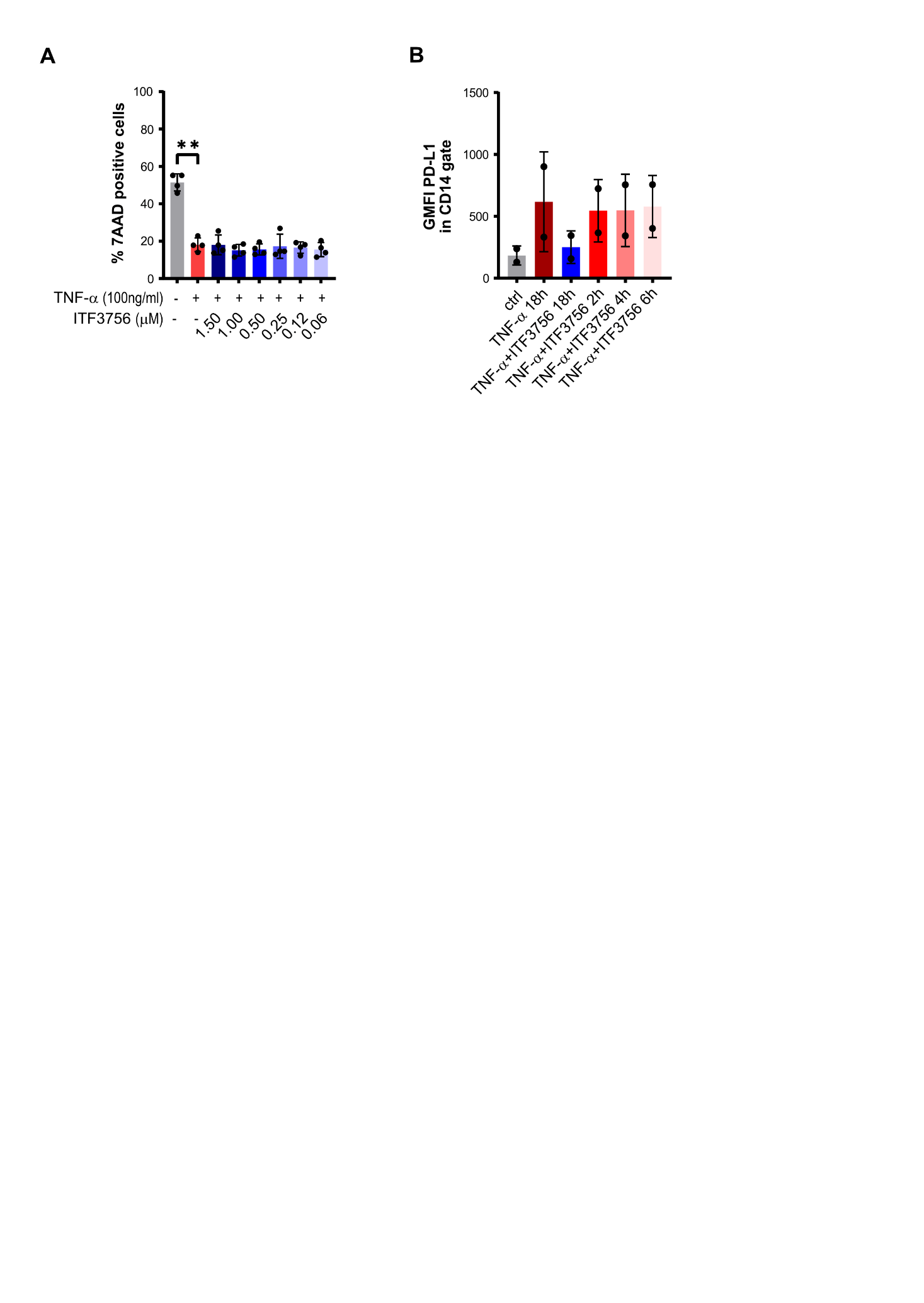


**Supplementary Figure 1**. **ITF3756 does not induce any cytotoxic effect on activated monocytes. TNF-α washout at different time point**

**A)** Human purified monocytes were treated for 2h with different concentration of ITF3756 (1.5μM-0.062μM) and then stimulated with TNF-α (100ng/ml) ON. Graph shows the percentage of 7ADD positive cells. Values on the graphs are expressed as mean±SD. n=4. P-values were calculated by one-way ANOVA test followed by Dunnetts multiple comparison test. *p<0,05, **p<0,001, ***p<0,0005 **B)** Human monocytes were stimulated with TNF-α (100ng/ml) for 2h, 4h, 6h, and 18h. After 2h, 4h, and 6h medium was removed and replaced with fresh medium without the cytokine. Expression of PD-L1 was analyzed for all conditions at 18h. Graph shows the geometric mean fluorescence intensity (GMFI) of PD-L1 at different time points. Values on the graphs are expressed as mean±SD. n=2.

**Supplementary Figure 2**

**A**

**** ****

**
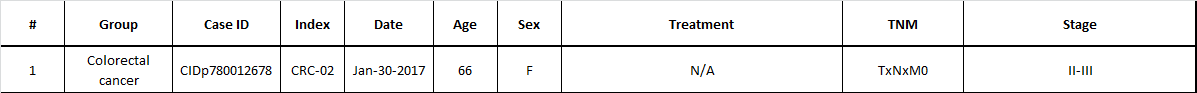
**

**B**

**Supplementary Figure 2. ITF3756 downregulates PD-L1 in human PBMC from cancer patients**

Human PBMC of cancer patients were treated for 2h with ITF3756 (1μM) and then stimulated with TNF-α (100ng/ml) ON. A) PBMC collected from patient with colorectal cancer. Percentage of PD-L1 positive cells and expression of PD-L1 measured as the fluorescence intensity geometric mean GMFI in the gate of CD14 positive cells. The values in the graphs represent the percentage of inhibition of PD-L1 in ITF3756 treated cells compare to TNF-α control. The table reports the patient data. B) PBMC collected from patient with breast cancer. Percentage of PD-L1 positive cells and expression of PD-L1 measured as the fluorescence intensity geometric mean GMFI in the gate of CD14 positive cells. The value in the graph represents the percentage of inhibition of PD-L1 in ITF3756 treated cells compare to TNF-α control. The table reports the patient data.

**Supplementary Figure 3**
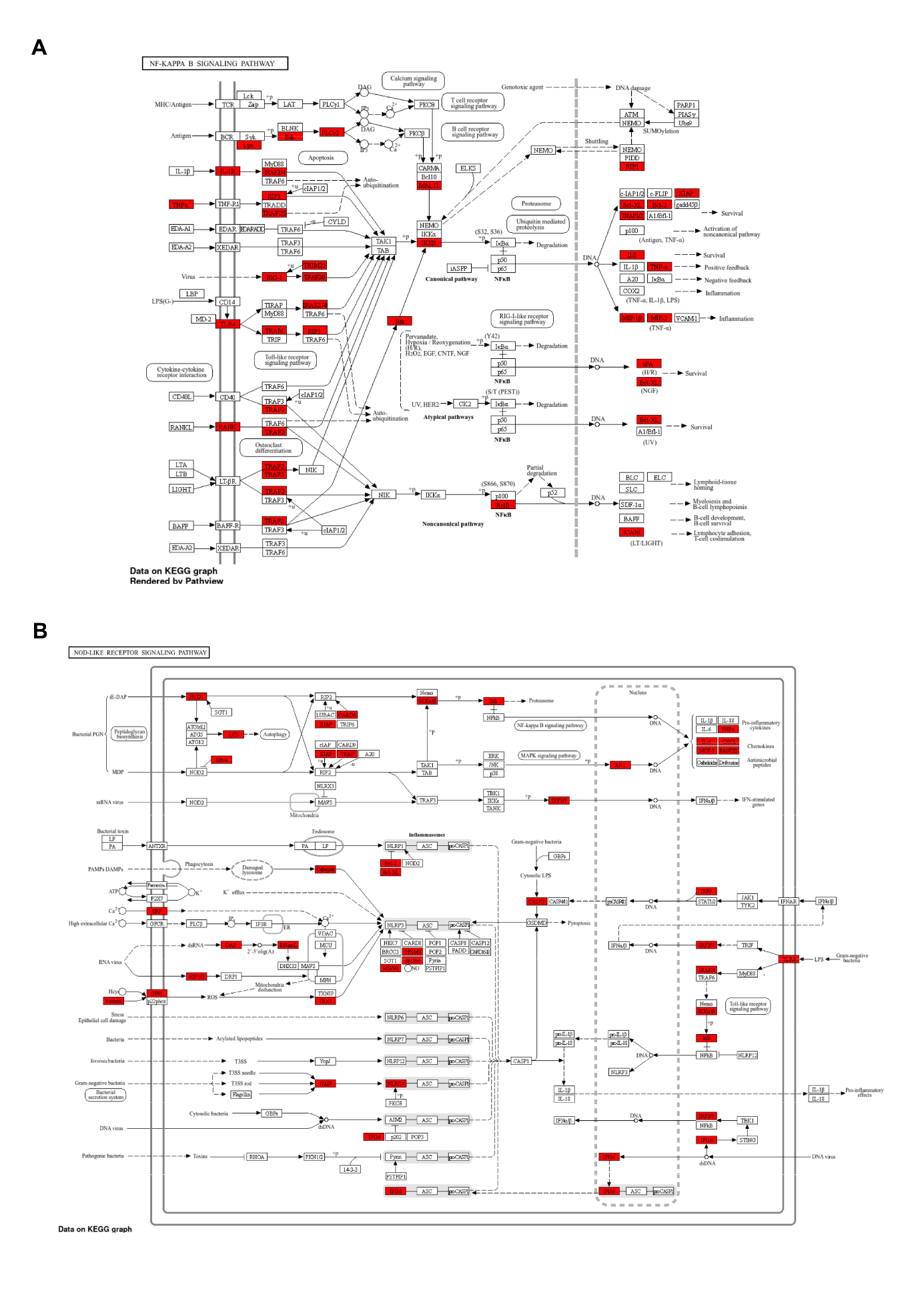


**Supplementary Figure 3. Transcriptomic analysis of TNF-α stimulated monocytes treated with ITF3756 shows that NF-kappa-B and NOD-like receptor signaling pathways are downmodulated by the HDAC6i**

Purified human monocytes were treated for 2h with ITF3756 (1μM) and then stimulated with TNF-α (100ng/ml) for 4h. **A)** KEGG pathway analysis and representation of the NF-kappa-B pathway; red boxes display the genes of the pathway downmodulated by ITF3756 treatment. **B)** KEGG pathway analysis and representation of the NOD-like receptor signaling pathway; display the genes of the pathway downmodulated by ITF3756 treatment.

**Supplementary Figure 4
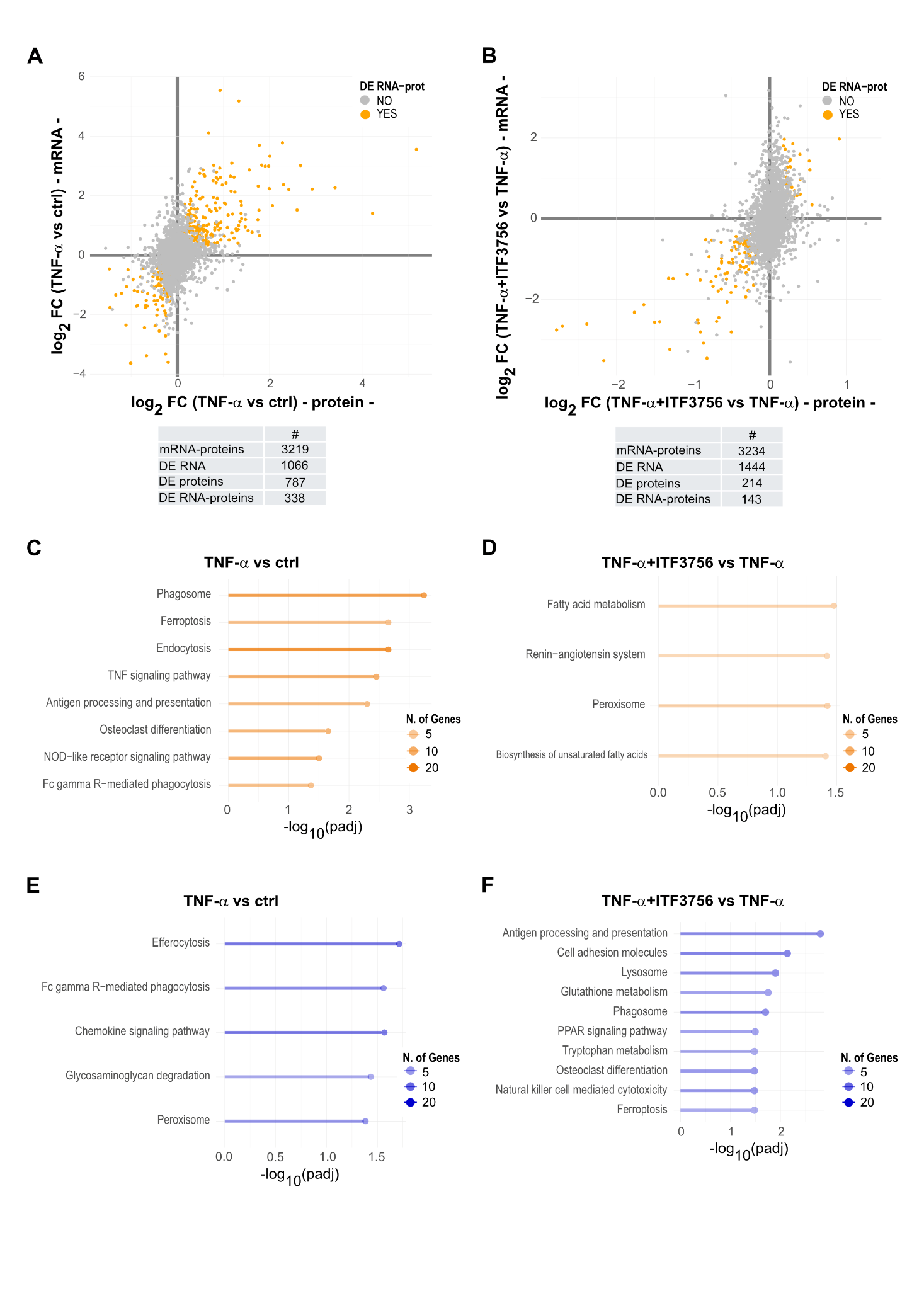
**

**Supplementary Figure 4. Correlation analysis of transcriptomic and proteomic data of TNF-α stimulated monocytes treated or not with ITF3756**

Purified human monocytes were treated for 2h with ITF3756 (1μM) and then stimulated with TNF-α (100ng/ml) for 4h or 18h for transcriptomic and proteomic analysis, respectively. **A)** Correlation analysis performed in TNF-α-stimulated monocytes. FC stands for fold changes in the graph. **B)** Correlation analysis performed in TNF-α-stimulated monocytes treated with ITF3756. FC stands for fold changes in the graph. **C and E)** Pathways analyses on significantly up- and down-regulated genes (orange and blue, respectively) in TNF- α stimulated monocytes were performed with EnrichR package. **D and F)** Pathways analyses on significantly up- and down-regulated genes (orange and blue, respectively) in monocytes stimulated with TNF- α and treated with ITF3756 were performed with EnrichR package.

**Supplementary Figure 5**

**
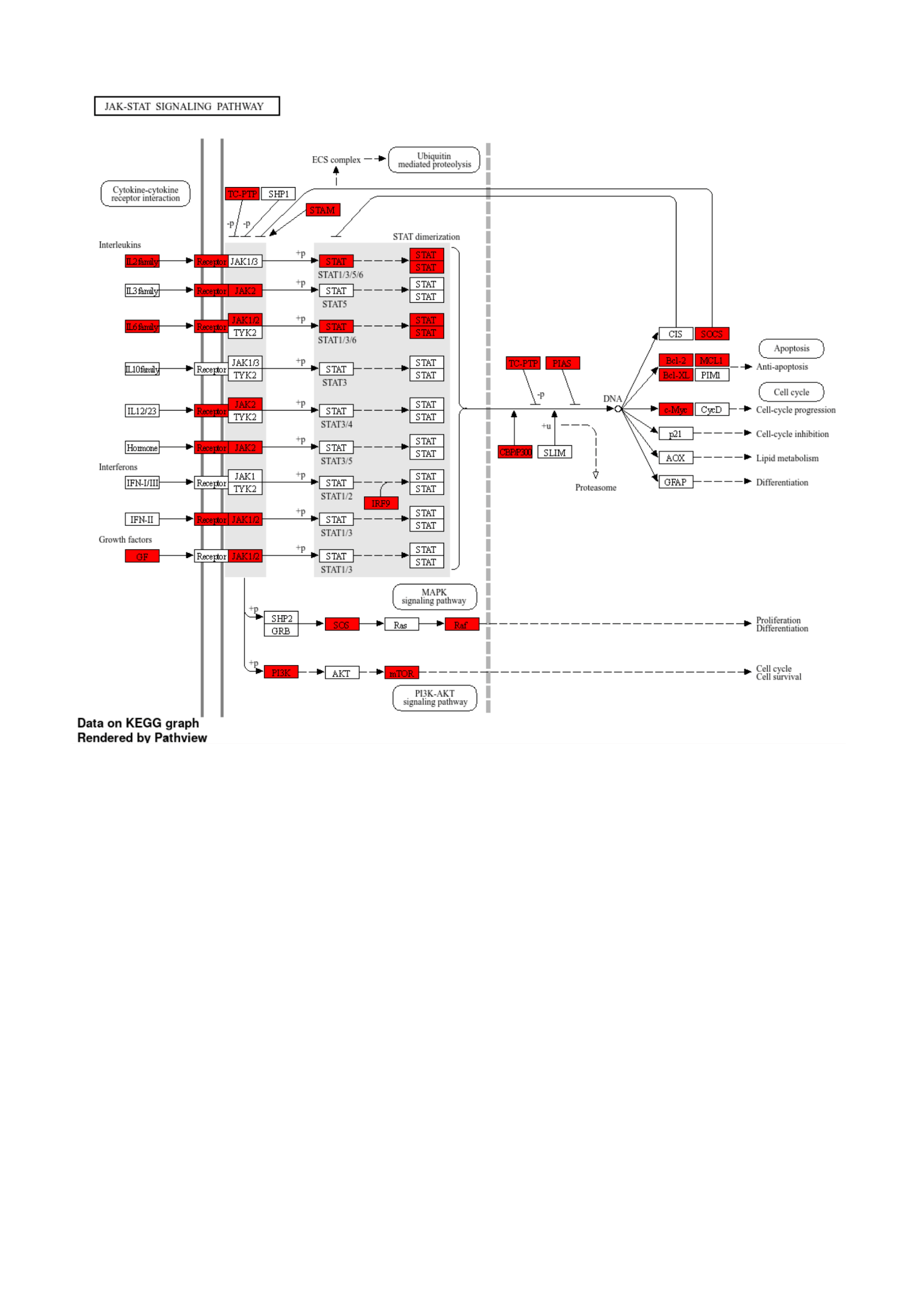
**

**Supplementary Figure 5.** **Transcriptomic analysis of TNF-α stimulated monocytes treated with ITF3756 shows that ITF3756 downmodulates JAK-STAT signaling pathway**

Purified human monocytes were treated for 2h with ITF3756 (1μM) and then stimulated with TNF-α (100ng/ml) for 4h. KEGG pathway analysis and representation of the JAK-STAT pathway; red boxes display the genes of the pathway downmodulated by ITF3756 treatment.

**Supplementary Table 1. Gene expression fold changes of *RELA*-target genes in monocytes treated with TNF-α and with the combination of TNF-α and ITF3756 relative to TNF-α alone.**

**Supplementary Table 2. Gene expression fold changes of *STAT1*-target genes in monocytes treated with TNF-α and with the combination of TNF-α and ITF3756 relative to TNF-α alone.**

**Supplementary Table 3. Gene expression fold changes of *IRF3*-target genes in monocytes treated with TNF-α and with the combination of TNF-α and ITF3756 relative to TNF-α alone.**
